# Gestational diabetes-related gut microbiome dysbiosis is not influenced by different Asian ethnicities and dietary interventions: a pilot study

**DOI:** 10.1038/s41598-024-60386-y

**Published:** 2024-04-29

**Authors:** Abhishek Gupta, Shiao Yng Chan, Rachel Toh, Jia Ming Low, Isabella Ming Zhen Liu, Su Lin Lim, Le Ye Lee, Sanjay Swarup

**Affiliations:** 1 Singapore Centre For Environmental Life Sciences Engineering (SCELSE), National University of Singapore, Singapore, Singapore; 2 Department of Obstetrics and Gynaecology, Yong Loo Lin School of Medicine, National University of Singapore, Singapore, Singapore; 3 Agency for Science, Technology and Research (A*STAR), Singapore Institute for Clinical Sciences (SICS), Singapore, Singapore; 4 Department of Neonatology, Khoo Teck Puat-National University Children’s Medical Institute, National University Hospital, National University Health System, Singapore, Singapore; 5 Department of Paediatrics, Yong Loo Lin School of Medicine, National University of Singapore, Singapore, Singapore; 6 Department of Dietetics, National University Hospital, National University Health System, Singapore, Singapore; 7 Foundation Healthcare Holdings, Singapore, Singapore; 8 Department of Biological Sciences, National University of Singapore, Singapore, Singapore; 9 NUS Environmental Research Institute, National University of Singapore, Singapore, Singapore

**Keywords:** Metabolic disorders, Molecular biology

## Abstract

Gut microbiome dysbiosis contributes to the pathophysiology of both gestational diabetes mellitus (GDM) and its associated adverse outcomes in the woman and offspring. Even though GDM prevalence, complications, and outcomes vary among different ethnic groups, limited information is available about the influence of ethnicity on gut microbiome dysbiosis in pregnancies complicated by GDM. This pilot prospective cohort study examined the impact of ethnicity on gut dysbiosis in GDM among three Asian ethnic groups (Chinese, Malay, Indian) living in Singapore, and investigated the potential modulatory roles of diet and lifestyle modifications on gut microbiome post-GDM diagnosis. Women with GDM (n = 53) and without GDM (n = 16) were recruited. Fecal samples were collected at 24–28- and 36–40-weeks’ gestation and analyzed by targeted 16S rRNA gene-based amplicon sequencing. Permutational multivariate analysis of variance (PERMANOVA) analysis was performed to evaluate differences between groups. Differentially abundant taxa were identified by DeSeq2 based analysis. Functional prediction was performed using the phylogenetic investigation of communities by reconstruction of unobserved states (PICRUSt2). Among women with GDM, gut microbiome from different ethnicities harbored common microbial features. However, among those without GDM, there was contrasting microbiome composition between ethnic groups. Microbial members such as *Collinsella, Blautia, Ruminococcus, Ruminococcus gnavus, Ruminococcus torques,* and *Eubacterium hallii groups* were differentially enriched (*p* < 0.05) in women with GDM compared to those without. Among women with GDM, no differences in alpha- and beta- diversity were observed when comparing 24–28 weeks’ samples with 36–40 weeks’ samples, a period covering intense dietary and lifestyle modification, suggesting an inability to modulate gut microbiota through classic GDM management. Women with GDM have a distinct gut microbiome profile which harbours common features across different Asian ethnic groups, consistent with the notion that specific microbes are involved in the pathogenesis of insulin resistance, pro-inflammatory conditions, and other metabolic dysregulation known to be present in GDM.

## Introduction

Gestational diabetes mellitus (GDM) is a metabolic disorder, defined as glucose intolerance that is first recognized during pregnancy^1^. Global data from 2020 estimated the prevalence of GDM to be 20%, depending upon the diagnostic criteria^2^. GDM increases the likelihood of many short- and long- term adverse outcomes for both women and neonates. GDM is associated with gestational hypertension and pre-eclampsia during pregnancy, and increased risks of type 2 diabetes mellitus (T2DM), cardiovascular disease, and other metabolic disorders after pregnancy^3–6^. Meanwhile, neonates of pregnancies complicated by GDM are at increased risk of macrosomia, hypoglycemia, jaundice, and later childhood obesity, T2DM and metabolic syndrome^7–9^. Several factors such as pre-pregnancy overweight or obesity, genetic predisposition, and family history of T2DM are strong risk factors for developing GDM and insulin resistance (IR)^10,11^. Therefore, it is necessary to find effective interventions to prevent and treat GDM; this involves first identifying specific pathogenic factors to target.

Gut microbiota has a crucial influence on human health, impacting the host immune system, metabolism, and endocrine system^12,13^. Moreover, a progressive natural alteration of gut microbiota from the non-pregnant state and through pregnancy has been reported but how this arises, and its implications, are not yet fully understood^14–17^. Nevertheless, such alteration in microbial composition during early to late pregnancy might be linked to the physiological hormonal changes or the consequent maternal metabolic shifts^14,16^. Recent evidence shows that imbalances in gut microbiota are associated with the pathogenesis of GDM, IR, and altered inflammatory responses^18–21^. However, it remains uncertain whether gut dysbiosis is a part of the cause, or a consequence of GDM progression, and when exactly this dysbiosis develops^22,23^. The contrasting and inconsistent results across studies comparing gut microbiome profiles between pregnant women with GDM and those without across the various regions of the globe have limited the acquisition of understanding and the development of effective microbiome-related intervention strategies for its prevention and treatment^24^. Even though optimal dietary intake and the adoption of a healthier lifestyle are first line treatments in GDM management, there are marked variations in their efficacy between individual women. Furthermore, these instituted measures may have a confounding effect on the GDM-microbiota-related profiles that are assessed after GDM diagnosis^25–28^. Hence, it is of utmost importance to have a better understanding about the role of gut microbiome in the pathophysiology of GDM. Furthermore, it has been observed that clinical characteristics, dietary intake, GDM outcomes, and its prevalence vary substantially among women from different ethnic communities such as Asian or Southeast Asian, Caucasian, African-American, and Hispanic groups^2,29,30^. To our knowledge, most studies which researched gut microbiome differences in GDM were limited to one ethnic group at a single time point in pregnancy^19–21,27,28,31–35^. Hence, an appreciation of the relationship between ethnicity and gut microbiome dysbiosis in GDM is limited. Therefore, it would be intriguing to understand the variability in gut microbiome profiles of women diagnosed with GDM among various ethnic groups, and identify the biomarkers or potential therapeutic targets associated with GDM to develop effective prevention and treatment strategies based on ethnicity.

The present pilot study was designed to understand the difference in gut microbiome profiles in GDM and non-GDM pregnancies, as well as to decipher the influence of ethnicity (Chinese, Malay, or Indian) on these differences. In addition, this study aimed to investigate the impact of dietary and lifestyle modifications on the gut microbiome of women with GDM comparing profiles soon after diagnosis with those towards the end of pregnancy. To fulfill these objectives, 16S rRNA gene-based targeted amplicon sequencing was performed and the results highlighted new insights that could be useful for the development of intervention strategies.

## Results

### Description of study participants

The characteristics of women with and without GDM are presented in Table 1. Maternal age, body mass index (BMI), height, and weight were matched between the two groups (all *p* > 0.05, respectively). Baseline fasting glucose was similar between those with and without GDM. Mean glucose values from oral glucose tolerance tests (OGTT) 1-h (10.0 vs. 7.3 mmol/L, *p* < 0.001) and 2-h (8.3 vs. 6.5 mmol/L, *p* < 0.001) were higher in women with GDM than in those without. Detailed descriptions of these characteristics within each ethnic group are presented in Supplementary Table S1.

**Table 1:** Characteristics of pregnant women with GDM (‘GDM’) and those without GDM (‘Controls’).

Variable	GDM		Controls		*p* value
	N^	Value	N^	Value	
Age (years)	53	32.83 ± 3.72	16	32.69 ± 2.91	0.87
Weight during first trimester (kg)	53	66.72 ± 12.01	14	63.257 ± 10.01	0.28
Height (m)	53	1.58 ± 0.07	16	1.6 ± 0.06	0.44
BMI during first trimester (kg/m^2^)	53	26.41 ± 4.35	16	24.65 ± 3.72	0.12
Fasting glucose (mmol/L)	53	4.61 ± 0.51	16	4.34 ± 0.35	0.08
Mean glucose at 1 h^#^ (mmol/L)	52	10.01 ± 1.36	16	7.5 ± 1.42	1.90E-06
Mean glucose at 2 h^#^ (mmol/L)	53	8.79 ± 1.03	16	6.32 ± 1.23	2.80E-07
GA during first trimester (weeks)	53	10.59 ± 3.76	15	8.47 ± 3.64	0.06
GA at delivery (weeks)	39	37.96 ± 1.61	16	38.63 ± 1.15	0.95
Weight at dietician’s counselling appointment soon after GDM diagnosis (kg)	39	67.35 ± 11.57	16	63.51 ± 8.79	0.19
Weight at end of pregnancy (kg)	39	69.77 ± 11.60	16	67.75 ± 9.35	0.5
Mean weight gain (kg)	39	7.65 ± 4.01	16	9.11 ± 4.17	0.24
Average calorie intake per day^&^ (calories/day)	39	1146.86 ± 254.72	13	1328.09 ± 739.72	0.4
Average protein intake per day^&^ (g/day)	39	59.93 ± 17.55	13	64.52 ± 41.85	0.71
Average fat intake per day^&^ (g/day)	39	47.41 ± 15.83	13	48 ± 33.25	0.95
Average carbohydrate intake per day^&^ (g/day)	39	119.27 ± 34.04	13	161.17 ± 74.82	0.07
Average sugar intake per day^&^ (g/day)	39	26.08 ± 34.30	13	34.3 ± 21.58	0.21
Average calcium intake per day^&^ (mg/day)	39	454.79 ± 461.79	13	461.79 ± 280.40	0.94
Average fibre intake per day^&^ (g/day)	39	13.02 ± 14.34	13	14.34 ± 10.18	0.67

*BMI* body mass index; *Ca* calcium; *CHO* carbohydrates; *GA* gestational age at delivery.

^number providing data.

Data is presented as mean ± SD.

^#^Plasma glucose in a 75 g oral glucose tolerance test at 24–28 weeks.

^&^According to 3-day food diary in the last trimester after diet counselling.

### Microbiome profile in women with GDM versus those without, and its association with ethnicity

**Figure 1:**
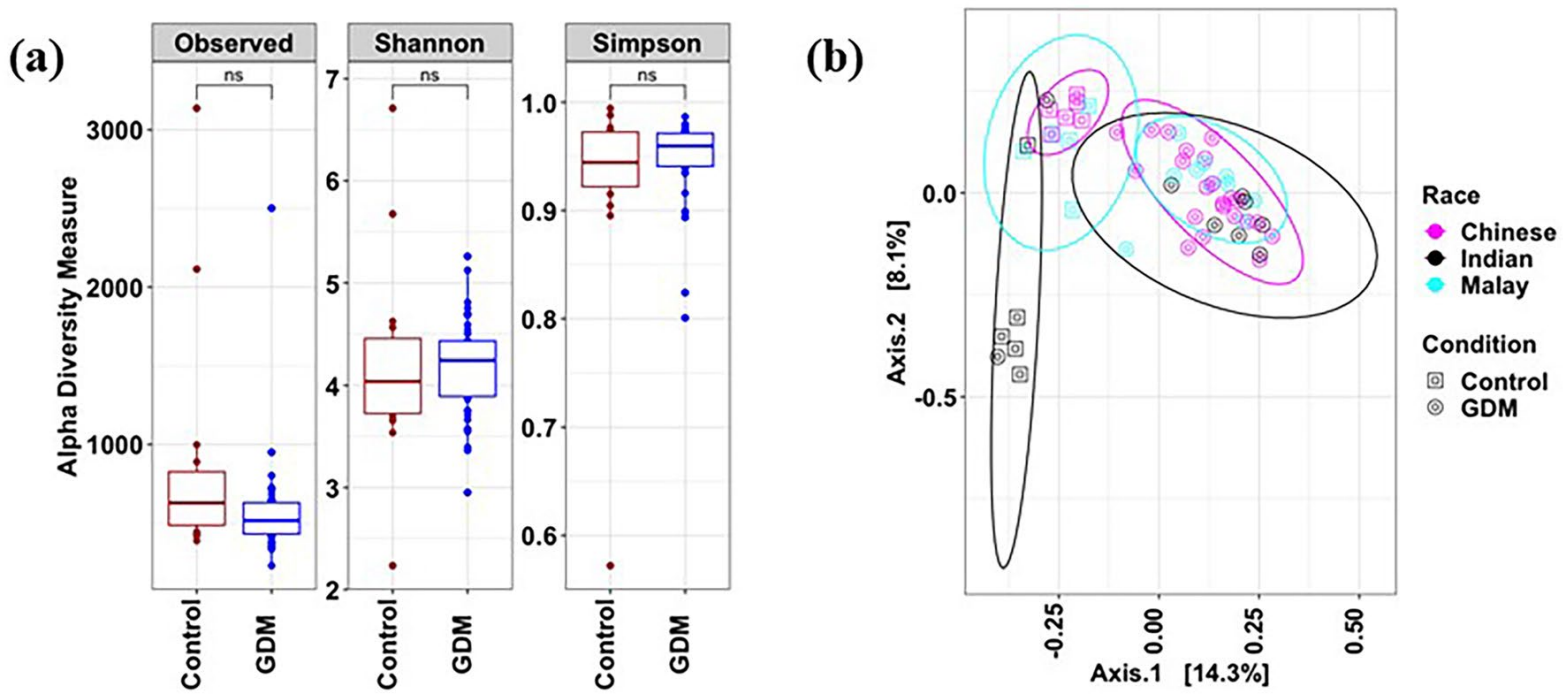
Microbiome composition of women with and without GDM. (**a**) α-diversity metrics of the gut microbiome; (**b**) PCoA-based analysis of the gut microbiome. *p < 0.05; **p < 0.01; ***p < 0.001; ns-non-significant.

Gut microbiome profiles of women with GDM were compared with those without using targeted 16S rRNA gene-based approach. Although no significant (*p* > 0.05) differences were observed with α-diversity measures (Simpson and Shannon indices), β-diversity was found to be significantly different between the women with GDM and without GDM [permutational multivariate analysis of variance (PERMANOVA), adjusted *p* < 0.05] (Fig. 1a,b). Notably, among women with GDM, no significant differences were observed between the different ethnic groups (Fig. 1b). Women with GDM from each of the three ethnic groups were noted to be different from their respective counterparts in the group without GDM, as detected through pairwise PERMANOVA analysis (Supplementary Table S2). Interestingly, amongst women without GDM, those of Indian ethnicity were found to be different from those of Chinese and Malay ethnicities, indicating that ethnicity does influence gut microbiome among women without GDM.

**Figure 2:**
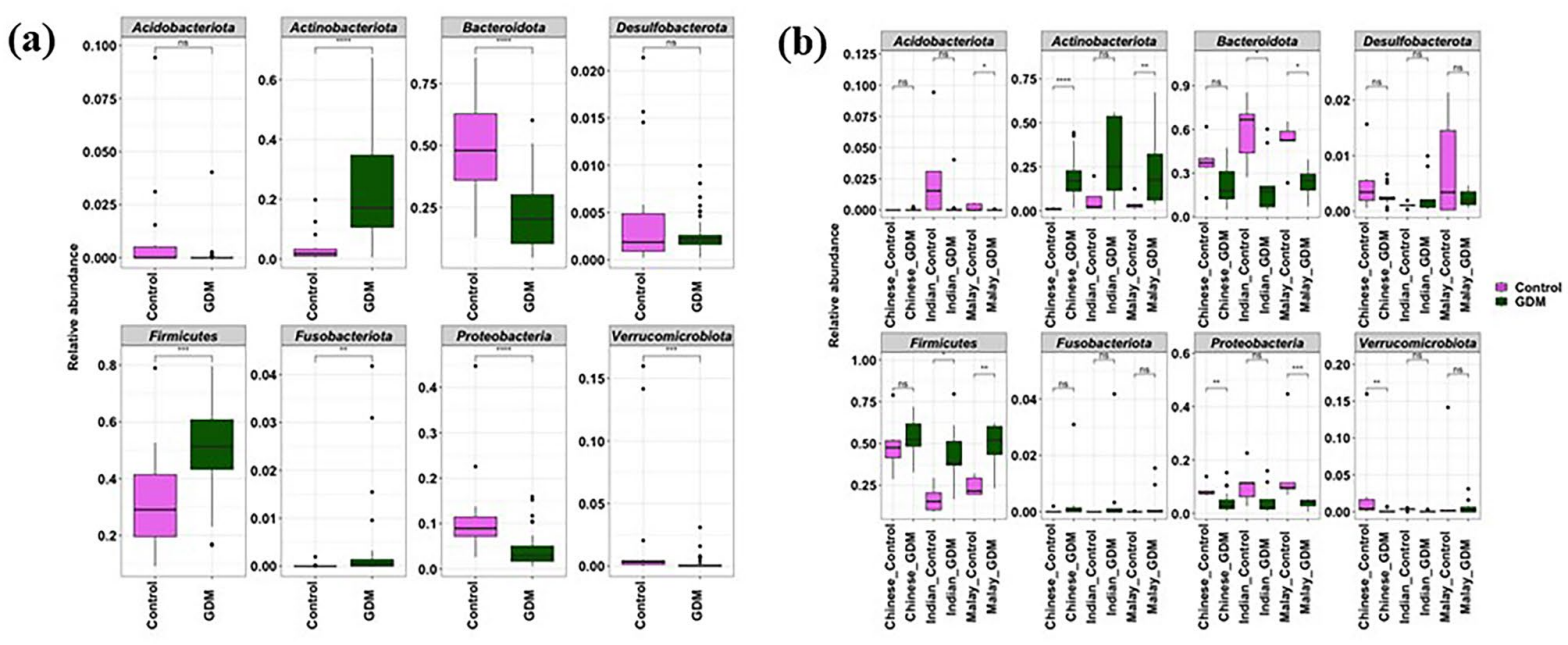
Change in bacterial composition between women with and without GDM across the three ethnic groups. (**a**) bacterial composition at phylum level in women with and without GDM; (**b**) bacterial composition at phylum level in the different ethnic groups. * p < 0.05; ** p < 0.01; *** p < 0.001; ns-non-significant.

To understand the microbiome profile difference between the two states (GDM and non-GDM), we looked further into the amplicon data. There was a distinct relative abundance of various bacterial phyla with a higher *Firmicutes* to *Bacteroidota* (F/B) ratio and a lower *Bacteroidota* to *Actinobacteriota* (B/A) ratio in women with GDM compared to those without. *Bacteroidota* (Wilcoxon test, *p* < 0.05), *Proteobacteria* (Wilcoxon test, *p* < 0.05), and *Verrucomicrobiota* (Wilcoxon test, *p* < 0.05) members predominated in women without GDM (Fig. 2a). On the contrary, *Actinobacteriota* (Wilcoxon test, *p* < 0.05), *Firmicutes* (Wilcoxon test, *p* < 0.05) and *Fusobacteriota* (Wilcoxon test, *p* < 0.05) were enriched in women with GDM (Fig. 2a). Within each ethnicity, a similar trend was noted between those with GDM and those without. Women of Indian and Malay ethnicities with GDM showed significantly (Wilcoxon test, *p* < 0.05) higher abundance of *Firmicutes* compared to those without GDM from the same ethnicities (Fig. 2b). Likewise, women of Chinese and Malay ethnicities with GDM showed significantly higher abundance of *Actinobacteria* (Wilcoxon test, *p* < 0.05) and lower abundance of *Proteobacteria* (Wilcoxon test, *p* < 0.05) compared to those without GDM from the same ethnicities (Fig. 2b). In addition, a significant decrease in the relative proportion of *Bacteroidota* (Wilcoxon test, *p* < 0.05) members was observed in women of Malay and Indian ethnicities with GDM, compared with their respective counterparts without GDM.

**Figure 3:**
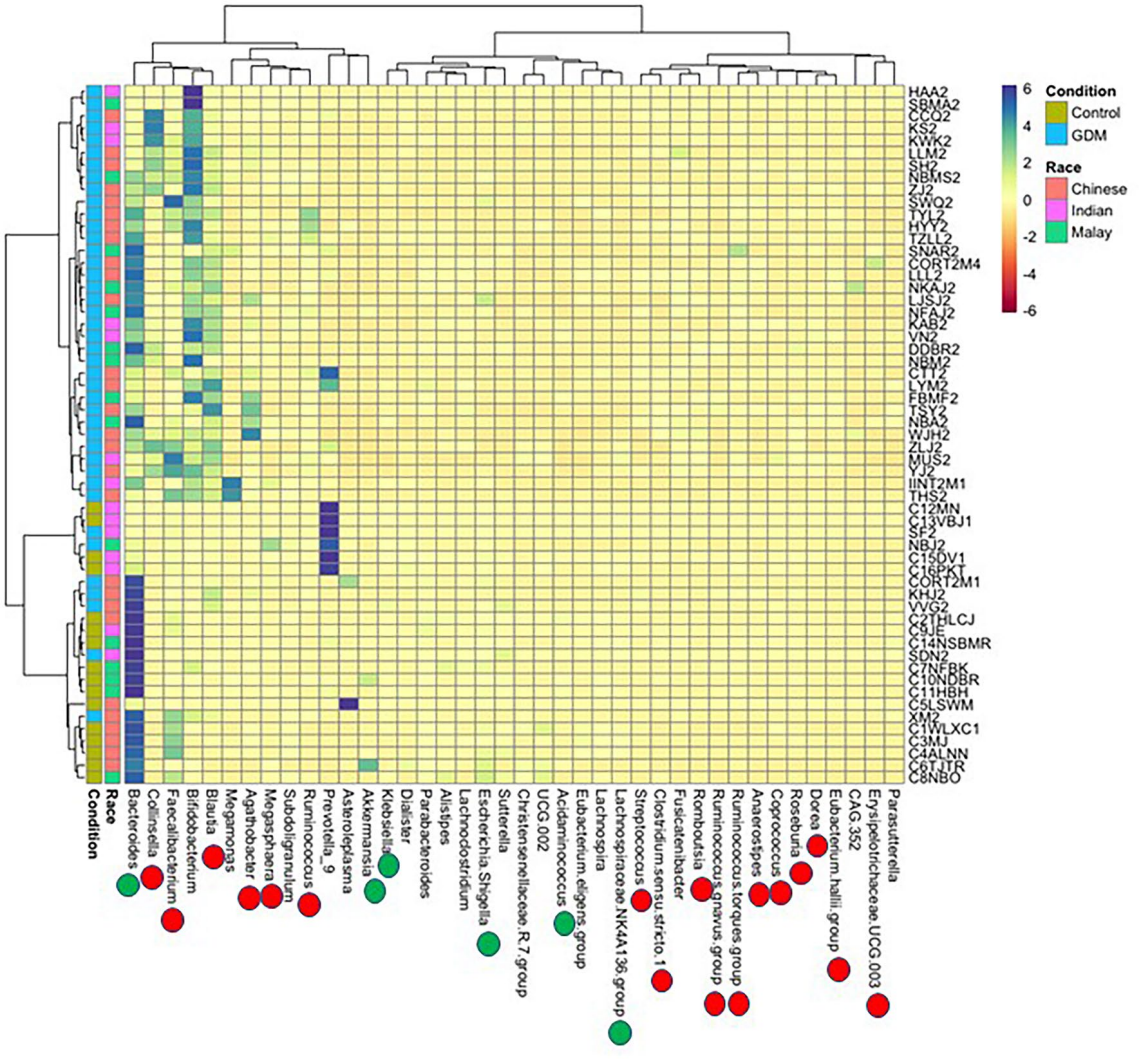
Heatmap based hierarchical clustering based on Bray Curtis dissimilarity matrix and Wald test of bacterial taxa and other parameters. Red dots represent the taxa which are significantly (Wilcoxon test, *p* < 0.05) abundant in women with GDM, while green dots represent the taxa significantly (Wilcoxon test, *p* < 0.05) abundant in women without GDM. The heatmap was generated using pheatmap package v1.0.12 ^77^.

Several differences were also detected in the gut microbiome profile of women with GDM compared to those without GDM at genera-level. Heatmap-based hierarchical clustering showed distinct visual clustering, separating the microbiome profiles of those with GDM from those without based on Bray–Curtis dissimilarity matrix (Fig. 3). However, clear cut separation based on ethnicity among women with GDM were not observed. More interestingly, bacterial genera such as *Collinsella, Blautia, Bifidobacterium, Dorea, Roseburia, Coprococcus, Anaerostipes, Ruminococcus gnavus group, Ruminococcus torques group, Eubacterium hallii group, Romboutsia, Fusicatenibacter, Clostridium *sensu stricto* 1, Agathobacter, Ruminococcus,* and *Megasphaera* were highly abundant in women with GDM as detected through pairwise Wilcoxon test (*p* < 0.05). In contrast, *Akkermensia, Bacteroides, Acidaminococcus, Escherichia-Shigella, Klebsiella,* and *Lachnospiraceae NK4A136 group* predominated (Wilcoxon test, *p* < 0.05) in those without GDM.

### Differentially abundant taxa associated with GDM condition

**Figure 4:**
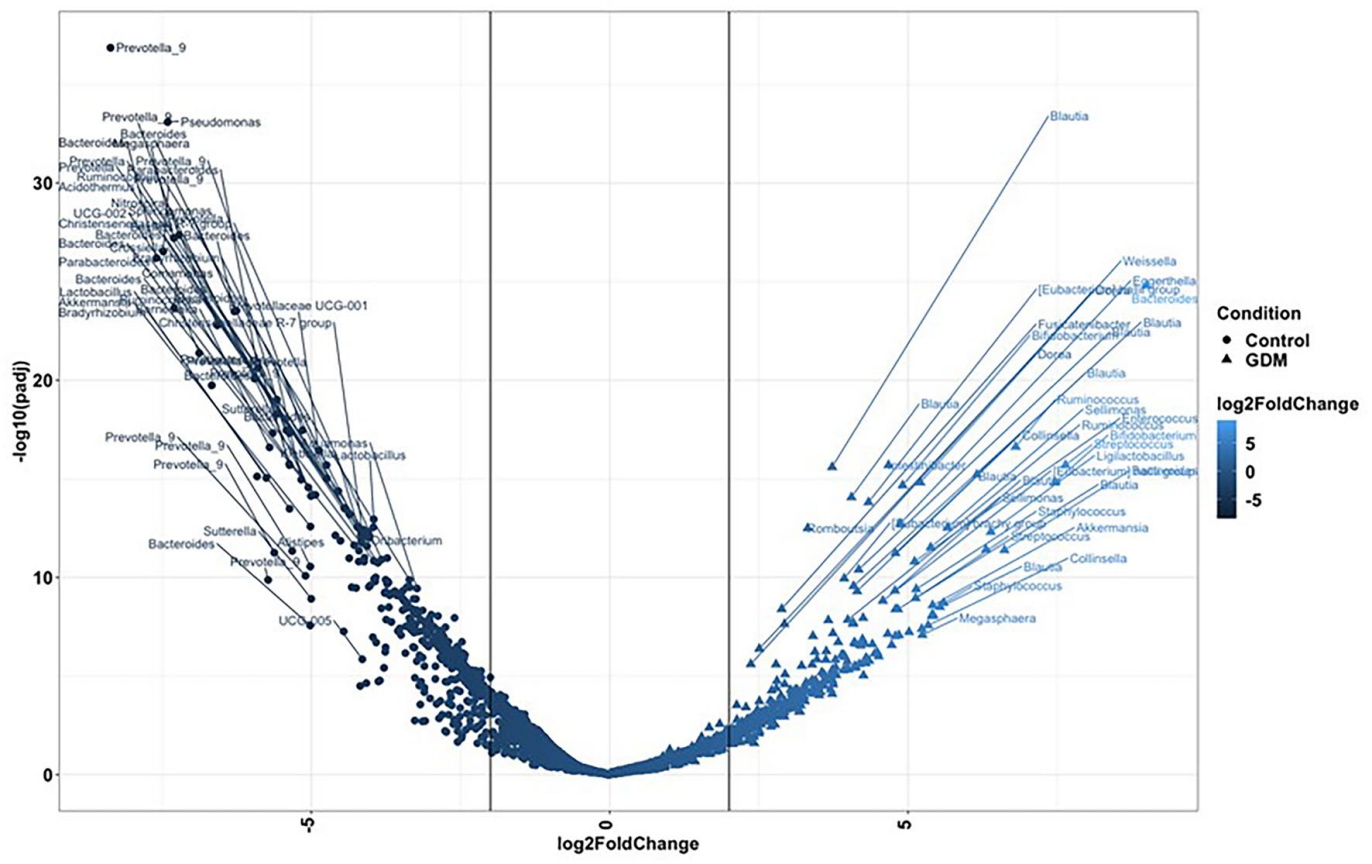
Volcano plot represent the differentially abundant ASVs (*p. adjusted* < 0.001) identified from DeSeq2-based analysis between women with and without GDM.

In relation to the microbes associated with the GDM state, DeSeq2-based analysis identified several differentially abundant microbial amplicon sequence variants (ASVs) in women with and without GDM. Various ASVs were found to be enriched in each state (GDM and non-GDM) as evident from the volcano plot (Fig. 4). ASVs affiliated to *Ruminococcus, Blautia, Collinsella, Bifidobacterium, Streptococcus, Sellimonas, Staphylococcus, Weissella, and Ligilactobacillus* were enriched (log2 fold change > 5; *p* < 0.001) in women with GDM. However, ASVs associated with *Bacteriodes, Pseudomonas, Prevotella 9, Megasphaera, Lactobacillus, Sutterella, Akkermansia, and Parabacteroides* were differentially enriched or abundant (log2 fold change > 5; *p* < 0.001) in those without GDM. A detailed list of differentially abundant ASVs is provided in Supplementary Table S3. To further explore the data and understand the microbial dynamics based on ethnicity, DeSeq2-based analysis was performed on each ethnic group separately to identify differentially abundant ASVs (representing taxa) in women with and without GDM (Supplementary Fig. S1). Each ethnic group demonstrated distinct differentially abundant ASVs along with some common ASVs in GDM and non-GDM states. Of note, however, ASVs affiliated to *Blautia, Ruminococcus, Bifidobacterium, Eubacterium hallii group, Streptococcus, Roseburia,* and others were enriched in women with GDM in all the three ethnic groups (Supplementary Fig. S1). Furthermore, a few additional ASVs were also enriched in either one or two ethnic groups. ASVs belonging to *Sellimonas, Intestinibacter, Collinsella, Ruminococcus torques group,* and others were differentially abundant in Chinese and Indian women with GDM (Supplementary Fig. S1a and c), whereas *Eggerthella, Sutterella, Weissella, Lachnoclostridum, Prevotella 9,* and others were enriched in Malay and Chinese women with GDM (Supplementary Fig. S1a,b).

### Predicted functional metabolic profiles of women with and without GDM

The predicted functional pathways of the gut microbiome of women with and without GDM were determined through phylogenetic investigation of communities by reconstruction of unobserved states (PICRUSt2) analysis. The results revealed that the predicted functional profile of gut microbiome of women with GDM was different from that of women without GDM (Supplementary Fig. S2a). The pathway enrichment analysis of the predicted functional KEGG categories/metabolisms showed an enrichment of various pathways of carbohydrate metabolism (C5-branched dibasic acid metabolism, pentose phosphate pathway, and starch and sucrose metabolism), amino acid metabolism (valine, leucine and isoleucine biosynthesis, cysteine and methionine metabolism, phenylalanine, tyrosine, and tryptophan biosynthesis, and lysine biosynthesis), cofactor and vitamins (pantothenate and CoA biosynthesis and thiamine metabolism), nucleotide metabolisms (purine metabolism and nucleotide excision repairs), and transporters (ABC transporters) in women with GDM (Supplementary Fig. S2b).

### Comparison of microbiome profiles of women with GDM at 24–28 weeks of gestation (just after diagnosis) with profiles at 36–40 weeks of gestation (last stage of pregnancy)

**Figure 5:**
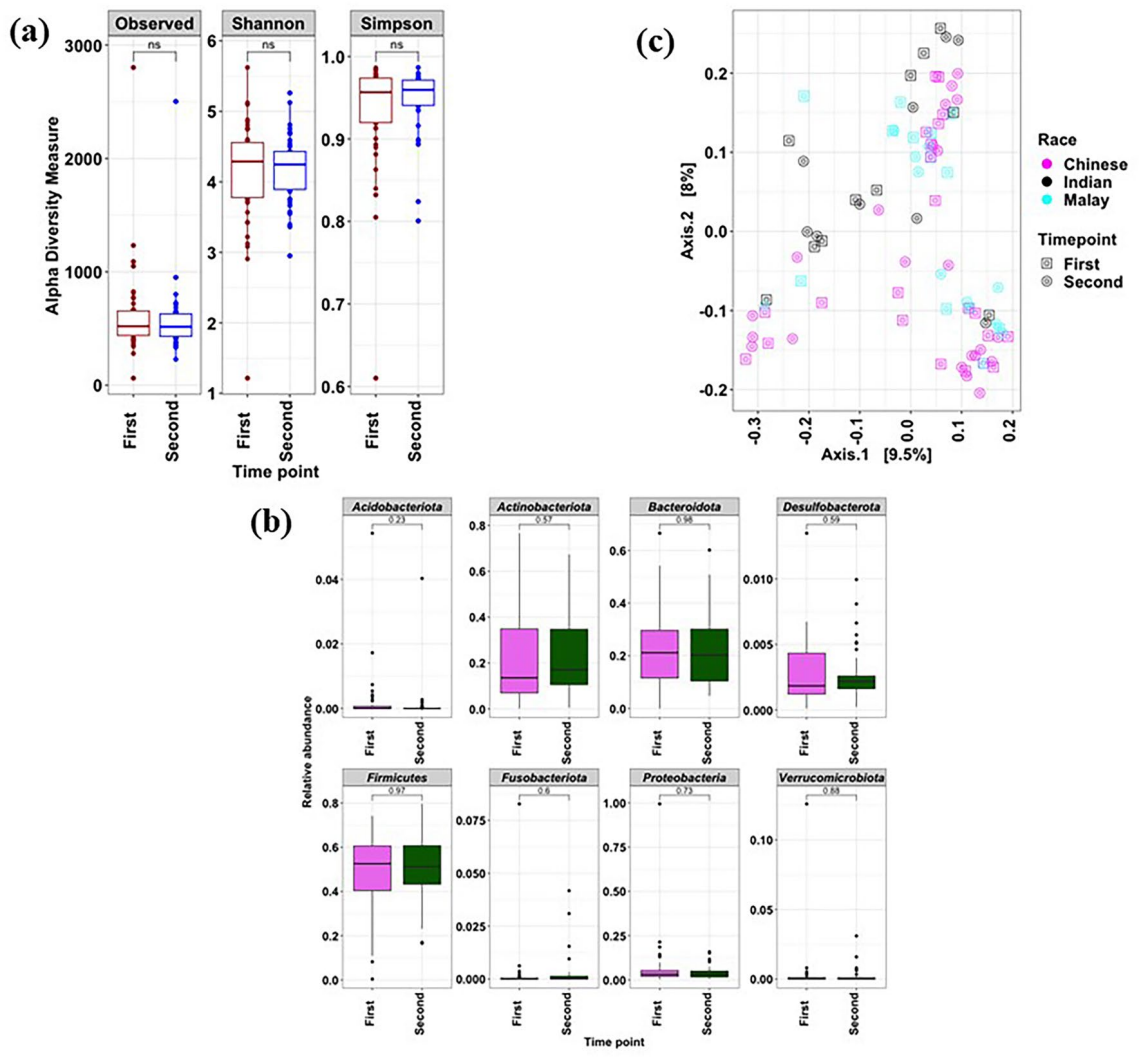
Microbial composition of women with GDM at two time points. (**a**) α-diversity metrics of the gut microbiome; (**b**) PCoA-based analysis of the gut microbiome between the two time points of women with GDM; (**c**) Bacterial composition of major phyla. * p < 0.05; ** p < 0.01; ***p < 0.001; ns-non-significant.

We probed for shifts in microbiome profiles of women with GDM from the time just after diagnosis till the last stage of gestation. α-diversity measures did not show significant differences (*p* > 0.05, Wilcoxon test) in the species diversity and richness between the two time points (Fig. 5a). *Firmicutes* was found to be the dominant member of the gut microbiome, followed by *Bacteroidota, Actinobacteriota,* and *Proteobacteria* (Fig. 5b). However, no significant difference (*p* > 0.05) was observed in the *Firmicutes* and *Bacteroidota* ratio between the two time points. Principal coordinate analysis (PCoA) yielded an overlapping microbiome pattern at the two time points (Fig. 5c). PERMANOVA analysis further confirmed that overall, there was no significant difference (*p* > 0.05) in the microbial composition of women with GDM between the two time points.

**Figure 6:**
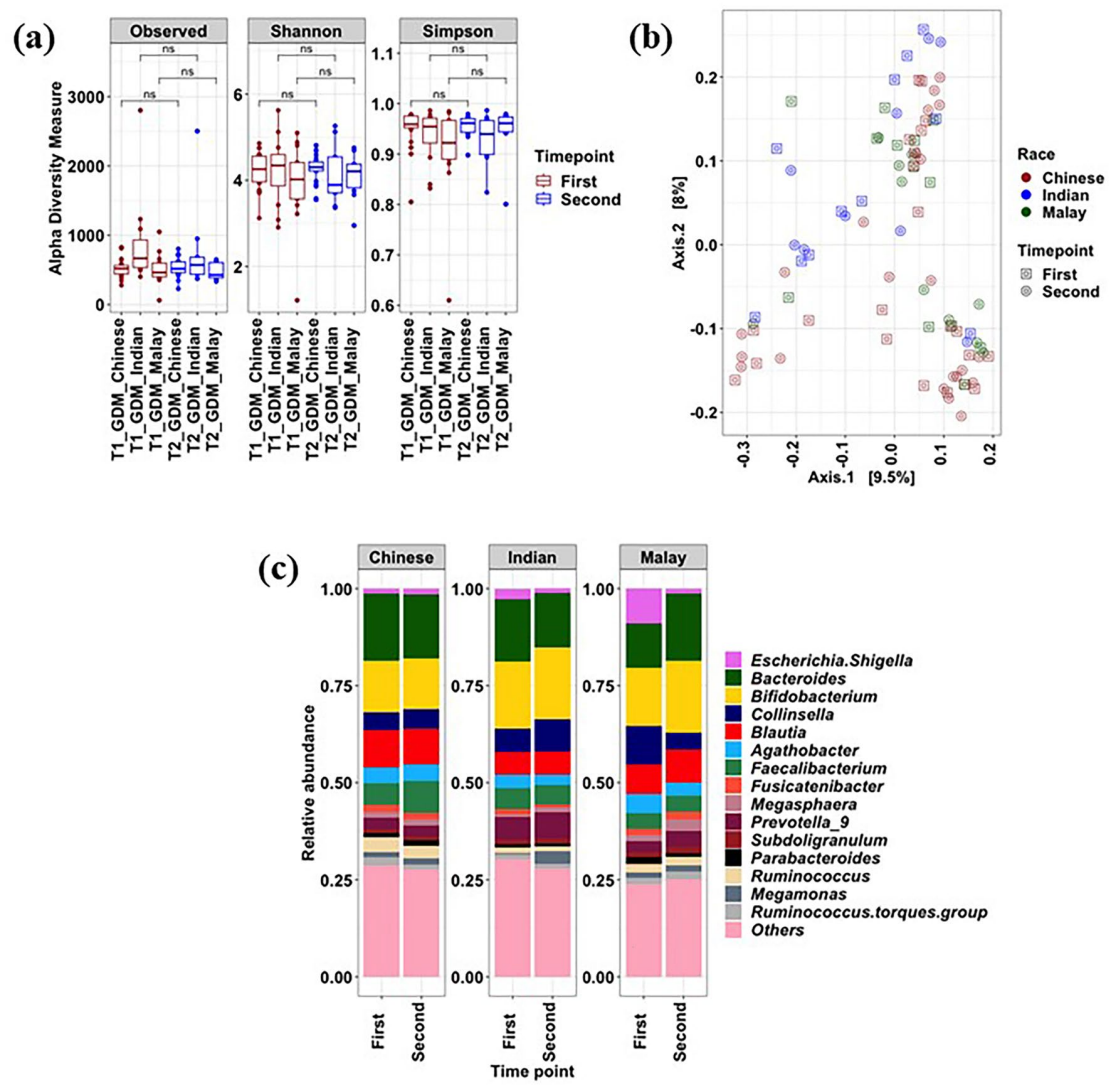
Microbial composition of women with at two time points based on ethnicity groupings. (**a**) α-diversity metrics of the gut microbiome; (**b**) PCoA-based analysis of the gut microbiome; (**c**) Bacterial composition of major genera. *p < 0.05; **p < 0.01; ***p < 0.001; ns-non-significant.

Among women with GDM, the three ethnic groups also did not show distinct microbiome profiles at both α- and β-diversity levels (Fig. 6a,b). Across the three ethnic groups, stool microbiome did not show significant changes (*p* > 0.05, Wilcoxon test) between the two time points in the abundance of *Firmicutes, Bacteroidota,* and *Actinobacteriota*. Bacterial genera such as *Bifidobacterium, Bacteroides, Collinsella, Blautia, Agathobacter, Prevotella_9, Megasphaera,* and *Ruminococcus,* which together constituted the major proportion of the gut microbiome in women with GDM (Fig. 6c).

### Association of plasma glucose and microbial taxa among women with GDM at the time of diagnosis (24-28 weeks)

To understand the association between BMI, glucose concentrations and microbial taxa, Spearman correlation-based analysis was performed (Supplementary Fig. S3). Fasting glucose positively correlated with weight and BMI at the time of OGTT, and negatively correlated with *Blautia, Ruminococcus, Acidaminococcus, Anaerostipes, Monoglobus,* and *Lachnoclostridium*. BMI was found to be negatively correlated with *Ruminococcus, Butyricicoccus, Anaerostipes*, and *Lachnoclostridium*, while one-hour glucose readings negatively correlated with *Eubacterium halli group, Ruminococcus gnavus group, Butyricicoccus,* and *Anaerostipes*. However, two-hour glucose readings were only negatively correlated with *Sutterella*, and positively correlated with *Dialister.*

### Dietary intervention and gut microbiome profile in women with GDM

**Figure 7:**
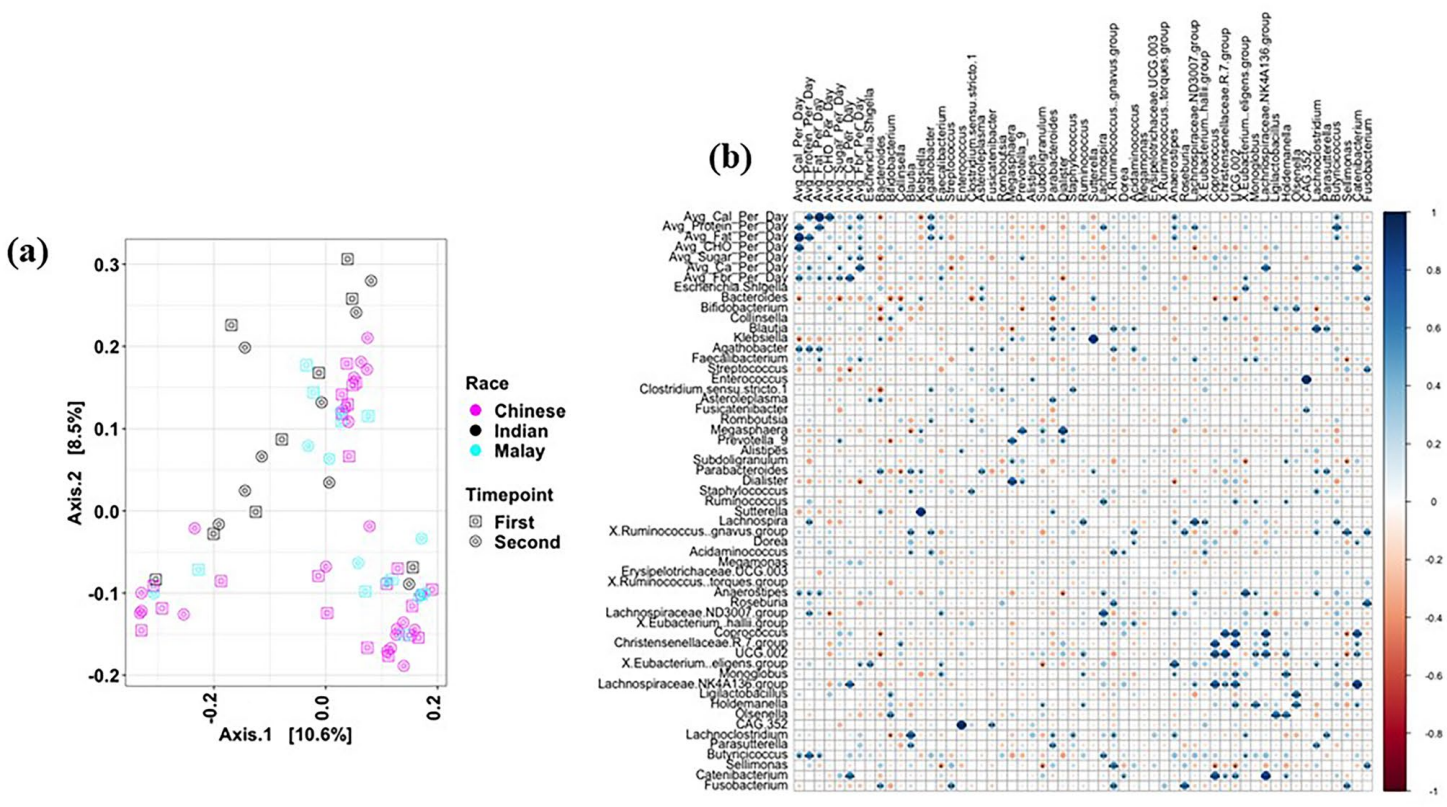
Changes in microbiome composition of women with GDM upon dietary interventions. (**a**) PCoA-based analysis of the gut microbiome of women with GDM after dietary interventions; (**b**) Heatmap-based Spearman correlation association between dietary intake and bacterial genera at 36–40 weeks of gestation. *p < 0.05; **p < 0.01; ***p < 0.001; ns-non-significant. Spearman correlation based heatmap was generated using corrplot package v0.92^79^.

All women with GDM underwent dietary counselling and lifestyle modifications to control glucose levels during pregnancy. In most cases, lifestyle interventions were deemed adequate. Analyses here were restricted to the 39 (72%) women with GDM who provided paired samples across both time points: at 24–28 weeks soon after GDM diagnosis, and at 36–40 weeks after dietary modification for 2–3 months. Overall, the microbiome profiles showed no significant differences between the two time points (PERMANOVA, *p* > 0.05) (Fig. 7a). Similar results were observed when women who underwent insulin therapy (n = 6) were excluded from the analysis. However, based on the dietary data, average protein intake per day was found to be positively correlated with *Agathobacter, Lachnospira, Anaerostipes, Butyricicoccus,* and *Lachnospiraceae ND3007 groups*, while *Fecalibacterium* and *Eubacterium eligens groups* were positively linked with average fibre per day (Fig. 7b). Likewise, *Fecalibacterium, Agathobacter, Anaerostipes,* and *Butyricicoccus* were found to be positively associated with average fat intake per day; only *Prevotella* was positively associated with average sugar intake per day (Fig. 7b).

## Discussion

GDM confers a broad spectrum of adverse health outcomes on both the mother and her offspring as a result of glucose intolerance during pregnancy^24,36^. It has been reported that the complications from GDM may vary with ethnicity; thus, specific ethnic groups that are at higher risk of GDM complications may benefit from more tailored education and strategies^37^. Moreover, dietary interventions and lifestyle modifications also play a vital role in the prevention of GDM^28^. In addition, recent reports have highlighted the significant role of microbial drivers in the pathogenesis of GDM^20–22,33^. Therefore, integration of the gut microbiome, dietary interventions, and ethnicity could be used as collective tool to develop effective prevention and treatment strategies for pregnant women to reduce the prevalence of GDM and its associated adverse outcomes. Hence, the present pilot study was designed to understand the impact of ethnicity and dietary interventions in pregnant women diagnosed with GDM, based on the dynamics of the gut microbiome. Results of the present pilot study demonstrate that women with GDM harbor distinct bacterial features compared to those without GDM. Similar gut microbial characteristics were observed in women with GDM across the three ethnic groups, indicating the enrichment of specific gut bacteria potentially involved in GDM pathophysiology along with other host-associated factors.

Our findings illustrate that GDM is associated with gut dysbiosis, with increased abundance of *Firmicutes* and *Actinobacteria*, and decreased amounts of *Bacteroides* and *Proteobacteria* populations in women with GDM compared to those without. These changes resulted in higher F/B and lower B/A ratios in women with GDM. Similar changes in the abundance pattern of these phyla have been reported in previous GDM microbiome studies, suggesting a role for gut dysbiosis in GDM pathophysiology^33,38^. An increase in F/B ratio in our study is in line with previous studies which reported a similar change in the ratio of *Firmicutes* and *Bacteroides* in GDM, obesity and other metabolic disorders; this increased ratio might underlie aggravative inflammation and insulin resistance^19,33,38,39^. However, some of the studies report conflicting results, where an increase in *Bacteroidota* members and a decrease in *Actinobacteria/Firmicutes* populations have been observed in GDM mothers, compared to euglycemic pregnant mothers^40,41^. Such differences in gut microbiome might be attributed to variations in several factors in study populations:—those that predate the pregnancy such as obesity, BMI, insulin sensitivity, adiposity, dietary habit, proinflammatory conditions, and ethnicity^24^, and those that arise in pregnancy such as weight gain and fetal factors (e.g., sex). Nevertheless, remodeling of microbial community structure in gut microbiome during normal pregnancy is a common process and has been linked to physiological hormonal and metabolic changes required for maternal adaptation to sustain a healthy pregnancy^14^. It is conceivable that the antenatal persistence of low-grade inflammation, increase in weight gain, and excessive adipose deposition, might collectively alter the gut microbiome metabolic responses to the pregnancy, hence promoting the development of insulin resistance and GDM^42,43^.

We also identified several microbial taxa that differentiated GDM from the non-GDM state; these could represent part of the gut dysbiosis event which may predate GDM development and be determined by the host-provided environment. Hence, it is crucial to understand their role in modulating the metabolic landscape of women who develop GDM, as it may lead to adverse outcomes. We observed that *Blautia, Collinsella, Eubacterium hallii group, Ruminococcus, Anaerostipes, Bifidobacterium, Ruminococcus gnavus group,* and *Ruminococcus torques group* were associated with GDM; these might be potential biomarkers for the diagnosis of GDM and therapeutic targets for development of preventive and treatment strategies. GDM-enriched microbial taxa, which have previously been reported in various GDM studies, are known to be associated with IR, obesity, T2DM, and low-grade inflammation^19,22,33,34,40,44^. Among them, *Collinsella* and *Blautia* are well-known microbial drivers for the diabetic state and have been associated with increased glucose and reduced insulin sensitivity^24,26,33,45,46^. Hence, the presence of *Collinsella* and *Blautia* in our sample of women with GDM compared to those without, supports the influence of this species on IR and potential development of GDM. Increased abundance of *Blautia* was previously associated with non-favorable metabolic profiles (i.e., unhealthy state) of individuals with high BMI^45^ and glucose intolerance^26,46^. However, Crusell et al.^33^ found that *Blautia* OTUs were associated with increased glucose and reduced insulin sensitivity, indicating their role in glucose metabolism and development or maintenance of GDM condition. Similarly, *Collinsella* is known to be associated with fasting plasma glucose and insulin levels^26,47^ and is sensitive to diet and weight loss^48,49^, supporting the influence of this bacteria on IR and the development of the GDM.

Moreover, together with these two main key players in diabetes development, *Eubacterium hallii group* (a member of *Lachnospiraceae*) is also involved in obesity and diabetes by promoting the dysfunction of islet β-cells^50,51^. *Eubacterium hallii group* can induce oxidative stress (through production of reuterin from glycerol) and cause cellular damage that has been implicated in GDM pathogenesis^52,53^. However, their roles in pathophysiology of GDM need further investigation. Furthermore, *Ruminococcus*, which is known to play a role in carbohydrate metabolism or even in short-chain fatty acid production, can cause excessive absorption of sugars by cells (increase energy harvest) leading to obesity or overweight^44^. Similarly, *Ruminococcus gnavus group* and *Ruminococcus torques group* have been previously reported in the GDM or diabetic state^44,54,55^. These members are proinflammatory and mucolytic in nature, resulting in decreased gut-barrier integrity, promotion of enrichment of opportunistic pathogens and, production of unhealthy metabolites during GDM^56,57^. A study conducted by Hu et al.^55^ found that insulin resistance, diabetes duration, stage, and medication alter the gut microbiota. This study further identified certain microbial taxa, such as *Eggerthella, Buytricicoccus, Romboutsia,* etc. which were associated with diabetic state, duration, and medication. Hence, increment of these genera in our GDM groups further support their plausible involvement in GDM development. However, further investigation is required to understand their role in human diseases associated with hyperglycemia, diabetes, and other metabolic disorders.

Our metabolic functional prediction based on 16S rRNA gene data further strengthens the role of these microbial taxa in GDM development and dysmetabolism. Our findings also demonstrate that various metabolic pathways or metabolism associated with carbohydrate, amino acid, cofactors and vitamins, nucleotide and transport are enriched in those with GDM, similar to other studies^21,25,41,58^. Increased pentose phosphate pathway in GDM mothers was associated with adiposity and insulin resistance as glucose-6-phosphate dehydrogenase enzymes promote dysfunction of pancreatic Beta-cell and apoptosis^59–61^. Pentose phosphate pathway are also known to be associated with purine metabolism, which play a significant role in impaired glucose metabolism^62^. Enrichment of amino acid metabolism was previously reported from GDM mothers, which are involved in insulin receptor signalling and glucose metabolism, indicating the importance of metabolomic interactome in diabetes or GDM development^63^.

The prevalence of GDM and outcomes varies by ethnicity^30^, with Asians and Pacific Islanders demonstrating a higher prevalence than White Caucasians. Several studies relating gut microbiome with GDM have been conducted worldwide including in Chinese^20,21,34,35^, Finnish^14,27,64^, Australian^32^, Brazilian^19^, German^31^, and Nordic^28,33^ populations. However, these studies either comprised only of one ethnic group, or multiple ethnic groups but did not investigate the impact of ethnicity on GDM microbiome. In our study, we tried to understand the influence of ethnicity on gut dysbiosis and its association with GDM. Our findings suggest that women with GDM harbor a similar microbiome profile among the three major Asian ethnic groups (Chinese, Malay, and Indian) in Singapore, and this profile of gut dysbiosis in GDM indicate that the enrichment of microbial taxa associated with carbohydrate metabolism, insulin resistance, obesity, and weight gain. There was a lack of difference in β-diversity among women with GDM from the different ethnic groups. Interestingly, women without GDM from different ethnic groups showed contrasting microbiome, indicating that among euglycemic pregnancies, ethnicity does influence gut microbiome profiles. This further supports the postulation that specific microbial taxa in women with GDM, which are common across ethnicities, could alter the physiological metabolic landscape and promote pathogenesis of GDM and promote IR during the maternal metabolic adaptation to pregnancy. This postulation is also consistent with recent reports that transplanted stool from women with GDM into germ-free mice could induce maternal gut dysbiosis that affects their offspring, which showed higher body weight and blood glucose levels compared with controls^23^. However, we cannot entirely disregard the possibility that some of the microbiome characteristics associated with GDM may have arisen as a result of the disease development, or that pre-existing pre-pregnancy metabolic vulnerabilities could have led to the gut dysbiosis observed.

In addition, our study also showed that dietary interventions and lifestyle modifications following GDM diagnosis did not change the gut microbiome profile. Such interventions have been demonstrated to be effective in promoting normoglycemia in a sizeable proportion of women with GDM in many studies^65–67^. This suggests that other mechanisms may regulate glycemia and overcome the postulated microbiome-driven effects that promote hyperglycaemia. Previous studies which similarly reported a lack of change in microbiome following dietary interventions/lifestyle modifications suggest that modulation of gut microbiota is inflexible due to the GDM state, since such interventions could clearly alter microbiome in euglycemic women^25–28^. It has been postulated that such a lack of plasticity towards dietary interventions in GDM maybe due to established gut dysbiosis or the limited opportunity for the dietary changes to establish a change in the microbiome over a relatively short time period of 2–3 months. Such a postulation of the lack of plasticity is also consistent with reports that interventions using probiotics supplements in GDM also failed to demonstrate a significant clinical effect^68–70^. A recent publication also showed that while the microbial community composition in GDM women remained unchanged after dietary intervention, inter-species co-abundance network was significantly altered^71^.

Limitations of our pilot study are the small sample size and few data points which may have led to missing the identification of other bacteria associated with GDM. Due to the lack of data on the gut microbiome profile of these pregnant women from the time of conception to the diagnosis of the GDM, we could not clearly demonstrate how the permanent resident microbes were enriched under the influence of metabolic and immunological changes in the host. This study also precluded investigation of links with GDM-related clinical outcomes. Additional collection of maternal stools in early pregnancy pre-OGTT diagnosis, as well as the inclusion of women with pre-existing diabetes, will allow us to better establish the chain of causation between gut microbiome evolution and development of hyperglycaemia during pregnancy. Further multi-omics-based longitudinal studies from preconception to the post-partum period from more diverse ethnicities around the world (either in native or immigrant populations) will help to determine the extent to which gut dysbiosis is driving the GDM pathophysiological process. Subsequently, meta-transcriptomics studies can also be performed to establish functionality and relate that to maternal glycaemia and other metabolic parameters as well as GDM-related clinical outcomes, and finally lead to the development of innovative therapeutic strategies.

## Conclusion

In conclusion, our findings suggest that the gut microbiome features of women with GDM are similar in all three ethnic groups despite clear ethnic differences among those without GDM; this strongly suggests a significant role in gut microbiome in the pathophysiology of GDM.

## Methods

### Recruitment of study participants

Ethical approval was obtained from the National Healthcare Group Domain Specific Review Board, Singapore (B2019/00064). All study procedures complied with the ethical guidelines of the Declaration of Helsinki. Pregnant participants were recruited between October 2019 and August 2021 from the National University Hospital, Singapore (NUH) antenatal clinic. Written informed consent was obtained from all participants. All participants had undergone a routine three-time-point 75 g oral glucose tolerance testing (OGTT) between 24 and 28 weeks of gestation as part of one-step universal screening for GDM at NUH. GDM was diagnosed using WHO (World Health Organization) 2013 criteria: fasting plasma glucose ≥ 5.1 mmol/L, or a 1-h glucose ≥ 10.0 mmol/L, or a 2-h glucose ≥ 8.5 mmol/L. Pregnant women who fulfilled the following criteria were recruited: (i) completed OGTT at 24–28 weeks; (ii) Chinese, Malay, or Indian descent; (iii) 25–40 years of age; (iv) body mass index at OGTT testing between 20 and 35 kg/m^2^; (v) no maternal active infection; (vi) not taken probiotic supplements or antibiotics in the 1 month prior to stool collection; and, (vii) no other significant maternal co-morbidities (hypertension, cardiac or renal disease). In total, 53 women with GDM (Chinese: n = 27, Malay: n = 15, Indian: n = 11) and 16 women without GDM (‘control’; Chinese: n = 6, Malay: n = 5, and Indian: n = 5) women were recruited.

### GDM management using nutritional and dietary trial therapy

All women with GDM received dietary counselling and lifestyle advice from dieticians and diabetes nurse educators. The team provided advice on dietary intake, carbohydrate portions, and how to achieve a balanced diet plan based on recommendations from the Academy of Nutrition and Dietetics for Gestational Diabetes^72^. Nutritional recommendations and calorie prescriptions were individualised, and took into consideration the patient’s body weight, weight gain, physical activity, fetal growth, as well as culture and usual cuisine that is commonly linked with her ethnicity. Adequate amounts of macronutrients to support pregnancy based on nutrition assessment and guidance from the recommended dietary allowance (RDA) were prescribed, including daily intakes of carbohydrates of 175 g or 42–60% of total calorie intake, a minimum protein of 71 g (or 1.1 g/kg body weight/day) and fiber of 28 g.

Individual nutrient intakes were derived from a 3-day food diary as recorded by the participants on the Nutritionist Buddy Diabetes (nBuddy Diabetes) app^73^. Participants were required to log their meals via the app, with the goal of keeping within the pre-set calorie and carbohydrate limits. Comparison of dietary intake between women with and without GDM was used to investigate the association of dietary modification with longitudinal change in gut microbiome. The average daily intake of carbohydrate, total calorie, sugars, protein, fat, calcium, and fiber was calculated based on 3 days from the electronic food diaries. These diaries were collected after they had been seen by the dieticians following diagnosis (if GDM) and prior to each stool collection. Women with GDM carried out self-blood glucose monitoring at 7 timepoints daily: before and after meals, and at bedtime. Insulin therapy was instituted if diet-control was insufficient to regulate their blood sugar. Weight and height at pregnancy booking in 1st trimester, at GDM diagnosis and at the last antenatal visit within a week of delivery were extracted from medical records or immediately post-delivery if the mothers did not have operations and were used to calculate BMI and weight change over the course of the study. Data pertaining to delivery and outcomes of neonates were also collected.

### Stool sample collection, DNA extraction, and 16S rRNA gene sequencing

Stool samples were collected from women with GDM at two time-points: 24–28 weeks of gestation (first time point soon after GDM diagnosis) and at 36–40 weeks of gestation (second time point just prior to delivery). However, only one time point (36–40 weeks of gestation) sampling was performed for women without GDM. Stool samples were collected in a sterile container and stored at − 80 °C till further processing. Total DNA was extracted from the stool samples using the CTAB/SDS method. DNA was further subjected to amplification of the V3-V4 regions (341F and 806R) and the amplified products were sequenced on the NovoSeq 6000 platform with 250X2 bp chemistry. Due to voluntary withdrawal of some participants prior to study completion and technical failures in sequencing, a total of 103 samples were finally included in analyses for the present study; these comprised 87 samples from women with GDM (first time point: n = 46, and second time point: n = 41) and 16 samples from women without GDM.

### 16S rRNA gene sequence analysis and statistics

Raw sequences obtained after sequencing were subjected to quality checking using FastQC^74^. Pre-processing and analysis were performed using DADA2 package v1.16.0^75^. Primers and low-quality bases were removed from the end of the reads using filter and Trim function of DADA2. Non-chimeric amplicon sequence variants were generated and subjected to taxonomic assignments using SILVA Database (silva_nr-99_v138.1_train_set.fa.gz). Both alpha and beta diversity metrics were generated by Phyloseq v3.4.2 R package^76^. Differences in alpha diversity metrics and relative abundance of bacterial taxa were tested between GDM and non-GDM pregnancies, or between ethnicities using pairwise Wilcoxon test. PCoA Principal coordinate analysis (PCoA) was performed with Bray–Curtis’s dissimilarity matrix to understand the difference in the community composition between the two conditions or based on ethnicities. Pairwise permutational multivariate analysis of variance (PERMANOVA) with false discovery rate (fdr)-adjusted *p* value was performed using the Bray Curtis dissimilarity matrix to assess the difference in beta diversity using pairwise adonis function. Beta dispersion analysis was performed using beta disper function to test the inter-individual variation. A heatmap was generated based on the major bacterial taxa using pheatmap package v1.0.12^77^. The hierarchical clustering was performed using Ward’s method and Bray–Curtis dissimilarity distance. Differential abundance analysis was performed using DeSeq2 package v1.38.3^78^ to determine the differentially enriched or depleted ASVs and/or biomarkers between the groups using Wald test and an adjusted *p* value filter of *p* < 0.01. Spearman correlation was calculated between bacterial taxa and clinical variables of GDM and non-GDM pregnancies using corrplot package v0.92^79^ and correlation was deemed statistically significant if *p* < 0.05. Various r packages such as ggplot2 v3.4.4^80^, RColorBrewer v1.1-3^81^, vegan v2.6-4^82^, and ggpubr v0.6.0^83^ were used for data visualization and other statistical tests. Functional prediction of gut microbiome profile was performed using the phylogenetic investigation of communities by reconstruction of unobserved states (PICRUSt2)^80^ followed by identification of statistically significant differentially abundant functional Kyoto Encyclopedia of Genes and Genomes (KEGG) categories/metabolisms between women with and without GDM using STAMP software^81^ implemented with Welch’s t-test and *p* values were adjusted by Bonferroni correction to minimize error.

### Ethics, consent and permissions

Ethical approval was obtained from the National Healthcare Group Domain Specific Review Board (NHG DSRB), Singapore (Reference no.: B2019/00064). Written informed consent was obtained from all participants.

### Supplementary Information

Supplementary Information.

## Data Availability

The raw sequences were submitted to the NCBI under Bioproject number PRJNA945212.
