# Supplementary material for: Gestational diabetes-related gut microbiome dysbiosis is not influenced by different Asian ethnicities and dietary interventions: a pilot study
Source: Sci Rep. 2024 Apr 29;14:9855. doi: 10.1038/s41598-024-60386-y (PMC11058859; doi:10.1038/s41598-024-60386-y)
Supplement: Supplementary file 1 — Supplementary Information. [file 41598_2024_60386_MOESM1_ESM.docx]

**Gestational diabetes-related gut microbiome dysbiosis is not influenced by different Asian ethnicities and dietary interventions: a pilot study**

Abhishek Gupta^1*^, Shiao Yng Chan^2,3^, Rachel Toh^4^, Jia Ming Low^4,5*^, Isabella Ming Zhen Liu^4^, Su Lin Lim^6^, Le Ye Lee^7^, Sanjay Swarup^1,8,9^

^1^ Singapore Centre For Environmental Life Sciences Engineering (SCELSE), National University of Singapore, Singapore

^2^ Department of Obstetrics & Gynaecology, Yong Loo Lin School of Medicine, National University of Singapore, Singapore

^3^ Singapore Institute for Clinical Sciences (SICS), Agency for Science, Technology and Research (A*STAR), Singapore

^4^ Department of Neonatology, Khoo Teck Puat-National University Children’s Medical Institute, National University Hospital, National University Health System, Singapore

^5^ Department of Paediatrics, Yong Loo Lin School of Medicine, National University of Singapore, Singapore

^6^ Department of Dietetics, National University Hospital, National University Health System, Singapore

^7^ Foundation Healthcare Holdings, Singapore

^8^ Department of Biological Sciences, National University of Singapore, Singapore

^9^ NUS Environmental Research Institute, National University of Singapore, Singapore

***Correspondence:**

Dr. Jia Ming Low

Department of Neonatology, Khoo Teck Puat-National University Children’s Medical Institute, National University Hospital, National University Health System, Singapore

Department of Paediatrics, Yong Loo Lin School of Medicine, National University of Singapore, Singapore

Email: [jia_ming_low@nuhs.edu.sg](mailto:jia_ming_low@nuhs.edu.sg)

Dr. Abhishek Gupta

Research Fellow, Singapore Centre For Environmental Life Sciences Engineering, National University of Singapore, Singapore

Email: [a_gupta7@nus.edu.sg](mailto:a_gupta7@nus.edu.sg)

**Supplementary Information**

**Supplementary Table S1** Characteristics of pregnant women with GDM (‘GDM’) and without GDM (‘Control’) grouped by ethnicity.

| **Variables** | **GDM** | **Control** | **GDM** | **Control** | **GDM** | **Control** |
| --- | --- | --- | --- | --- | --- | --- |
| Ethnicity | Malay | | Indian | | Chinese | |
| Age (years) | 31±4 (15) | 31±3 (5) | 33±4 (11) | 32±3 (5) | 34±3 (27) | 34±3 (6) |
| Weight during first trimester (kg) | 69.4±12.4 (15) | 64.2±8.5 (5) | 69.4±9.2 (11) | 67.7±12.8 (5) | 64.2±12.6 (27) | 56.5±4.9 (4) |
| Height (m) | 1.57±0.08 (15) | 1.59±0.07 (5) | 1.58±0.05 (11) | 1.59±0.05 (5) | 1.6±0.08 (27) | 1.62±0.05 (6) |
| BMI during first trimester (kg/m^2^) | 28±4.2 (15) | 25±1.8 (5) | 27.9±4.1 (11) | 26.9±5.1 (5) | 24.9±4.1 (27) | 22.5±2.7 (6) |
| Fasting glucose (mmol/L) | 4.84±0.53 (15) | 4.32±0.16 (5) | 4.77±0.52 (11) | 4.46±0.43 (5) | 4.5±0.48 (27) | 4.25±0.41 (6) |
| Mean glucose at 1h^#^ (mmol/L) | 10.53±1.31 (15) | 8.22±0.95 (5) | 10.35±0.71 (10) | 7.78±1.52 (5) | 9.61±1.47 (27) | 6.67±1.44 (6) |
| Mean glucose at 2h^#^ (mmol/L) | 8.64±0.96 (15) | 6.82±1.19 (5) | 8.58±1.58 (11) | 6.34±1.23 (5) | 8.96±0.77 (27) | 5.9±1.31 (6) |
| GA during first trimester (weeks) | 11±4 (10) | 8±3 (5) | 11±3 (9) | 8±6 (5) | 11±4 (19) | 10±1.9 (6) |
| GA at delivery (weeks) | 38±1.9 (15) | 39±1.14 (5) | 38±1.3 (11) | 39±1.09 (5) | 38±1.6 (26) | 38±1.16 (6) |
| Weight (kg) at dietician’s counselling appointment soon after GDM diagnosis | 70.7±13.8 (11) | 64.2±8.5 (5) | 71.6±8.1 (9) | 67.7±12.8 (5) | 63.4±10.8 (19) | 59.4±2.2 (6) |
| Weight at end of pregnancy (kg) | 73.2±14 (11) | 68.8±7.5 (5) | 74.5±8.5 (9) | 71.6±14.8 (5) | 65.6±10.3 (19) | 63.7±2.7 (6) |
| Mean weight gain (kg) | 6.8±4.2 (11) | 6.8±5.1 (5) | 9.4±4.3 (9) | 10.1±3.5 (5) | 7.3±3.7 (19) | 10.2±3.7 (6) |
| Average calorie intake per day^&^ (calories/day) | 1112±208 (10) | 1610±524 (5) | 1261±217 (6) | 891±633 (5) | 1132±282 (23) | 1586±1075 (3) |
| Average protein intake per day^&^ (g/day) | 52.3±14.9 (10) | 82.5±23.9 (5) | 56.7±11.8 (6) | 34.4±27 (5) | 64.1±19 (23) | 84.8±64.9 (3) |
| Average fat intake per day^&^ (g/day) | 42.9±14.3 (10) | 56.6±23.5 (5) | 55.6±10.6 (6) | 31.9±33.3 (5) | 47.2±17.7 (23) | 60.5±47.4 (3) |
| Average carbohydrate intake per day^&^ (g/day) | 130.6±21.6 (10) | 195.2±60 (5) | 132.8±34.3 (6) | 117.9±63.9 (5) | 110.8±36.9 (23) | 176.5±101.9 (3) |
| Average sugar intake per day^&^ (g/day) | 25.1±9.7 (10) | 40.0±23 (5) | 35.3±10.7 (6) | 29.8±18 (5) | 24.1±11.4 (23) | 32.3±30.4 (3) |
| Average calcium intake per day^&^ (mg/day) | 356.8±174.2 (10) | 476.4±194.6 (5) | 584.6±521.5 (6) | 362.7±271.6 (5) | 463.5±265.5 (23) | 602.6±440 (3) |
| Average fibre intake per day^&^ (g/day) | 10.2±3.8 (10) | 16.6±7.4 (5) | 15.2±8.7 (6) | 13.2±13.6 (5) | 13.7±7.3 (23) | 12.6±11 (3) |
| BMI: Body Mass index; Ca: Calcium; GA: Gestational age at delivery.  Data are presented as Mean±SD. The number in the brackets denote the no. of participants.  ^#^ Plasma glucose in a 75g oral glucose tolerance test at 24-28 weeks.  ^&^ According to 3-day food diary in the last trimester after diet counselling. | | | | | | |

**Supplementary Table S2** Pairwise PERMANOVA comparison of gut microbiome profile of GDM and non-GDM mother belonging to different ethnicity.

| **No.** | **Pairs** | **Df** | **SumsOfSqs** | **F.Model** | **R2** | ***P*-value** | ***P*-value (adjusted)** |
| --- | --- | --- | --- | --- | --- | --- | --- |
| 1 | Control_Malay vs Control_Indian | 1 | 0.618 | 1.975 | 0.198 | 0.026 | 0.035 |
| 2 | Control_Malay vs Control_Chinese | 1 | 0.385 | 1.250 | 0.122 | 0.108 | 0.125 |
| 3 | Control_Malay vs GDM_Chinese | 1 | 0.877 | 3.395 | 0.120 | 0.001 | 0.003 |
| 4 | Control_Malay vs GDM_Malay | 1 | 0.666 | 2.353 | 0.153 | 0.001 | 0.003 |
| 5 | Control_Malay vs GDM_Indian | 1 | 0.622 | 1.903 | 0.137 | 0.016 | 0.024 |
| 6 | Control_Indian vs Control_Chinese | 1 | 0.868 | 3.012 | 0.251 | 0.003 | 0.006 |
| 7 | Control_Indian vs GDM_Chinese | 1 | 1.352 | 5.378 | 0.177 | 0.001 | 0.003 |
| 8 | Control_Indian vs GDM_Malay | 1 | 1.141 | 4.235 | 0.246 | 0.001 | 0.003 |
| 9 | Control_Indian vs GDM_Indian | 1 | 0.893 | 2.861 | 0.193 | 0.008 | 0.013 |
| 10 | Control_Chinese vs GDM_Chinese | 1 | 1.056 | 4.192 | 0.139 | 0.001 | 0.003 |
| 11 | Control_Chinese vs GDM_Malay | 1 | 0.865 | 3.212 | 0.187 | 0.002 | 0.005 |
| 12 | Control_Chinese vs GDM_Indian | 1 | 0.801 | 2.596 | 0.166 | 0.004 | 0.008 |
| 13 | GDM_Chinese vs GDM_Malay | 1 | 0.262 | 1.054 | 0.034 | 0.368 | 0.394 |
| 14 | GDM_Chinese vs GDM_Indian | 1 | 0.358 | 1.347 | 0.044 | 0.073 | 0.091 |
| 15 | GDM_Malay vs GDM_Indian | 1 | 0.220 | 0.760 | 0.043 | 0.887 | 0.887 |

**Supplementary Table S3** Detailed list of differentially ASVs between GDM and control pregnant women identified through DeSeq2.

| **Genus** | **Condition** | **baseMean** | **log2FoldChange** | **lfcSE** | **stat** | **pvalue** | **padj** |
| --- | --- | --- | --- | --- | --- | --- | --- |
| Bifidobacterium | GDM | 3910.526 | 3.740 | 0.618 | 6.052 | 1.43E-09 | 6.70E-08 |
| Bifidobacterium | GDM | 342.860 | 3.788 | 0.719 | 5.266 | 1.39E-07 | 3.71E-06 |
| Collinsella | GDM | 2311.825 | 4.145 | 0.603 | 6.877 | 6.10E-12 | 4.93E-10 |
| Blautia | GDM | 2507.439 | 3.725 | 0.421 | 8.856 | 8.31E-19 | 2.51E-16 |
| Bifidobacterium | GDM | 1694.053 | 2.926 | 0.469 | 6.244 | 4.25E-10 | 2.28E-08 |
| Bifidobacterium | GDM | 2313.316 | 4.075 | 0.652 | 6.248 | 4.16E-10 | 2.24E-08 |
| Streptococcus | GDM | 143.386 | 7.636 | 0.859 | 8.888 | 6.23E-19 | 2.01E-16 |
| Agathobacter | GDM | 1382.158 | 2.283 | 0.544 | 4.193 | 2.75E-05 | 0.00038311 |
| Staphylococcus | GDM | 40.579 | 4.298 | 0.781 | 5.502 | 3.76E-08 | 1.19E-06 |
| Bifidobacterium | GDM | 126.509 | 7.455 | 0.864 | 8.628 | 6.24E-18 | 1.53E-15 |
| Streptococcus | GDM | 356.035 | 2.786 | 0.521 | 5.344 | 9.10E-08 | 2.53E-06 |
| Bifidobacterium | GDM | 266.860 | 2.474 | 0.602 | 4.112 | 3.91E-05 | 0.00051567 |
| Bifidobacterium | GDM | 38.298 | 4.314 | 0.793 | 5.443 | 5.24E-08 | 1.59E-06 |
| Clostridium sensu stricto 1 | GDM | 191.246 | 4.786 | 0.732 | 6.538 | 6.22E-11 | 4.06E-09 |
| Pseudomonas | Control | 48.333 | -7.406 | 0.579 | -12.793 | 1.78E-37 | 8.08E-34 |
| Fusicatenibacter | GDM | 910.860 | 2.880 | 0.440 | 6.543 | 6.04E-11 | 3.97E-09 |
| Romboutsia | GDM | 523.789 | 3.316 | 0.417 | 7.946 | 1.93E-15 | 3.19E-13 |
| Pseudomonas | GDM | 12.789 | 4.120 | 0.707 | 5.824 | 5.73E-09 | 2.28E-07 |
| Blautia | GDM | 616.035 | 4.170 | 0.574 | 7.260 | 3.87E-13 | 3.99E-11 |
| Bacteroides | GDM | 366.123 | 8.990 | 0.810 | 11.101 | 1.25E-28 | 1.61E-25 |
| Blautia | GDM | 578.772 | 4.049 | 0.481 | 8.412 | 4.03E-17 | 8.48E-15 |
| Prevotella_9 | Control | 640.895 | -4.181 | 0.870 | -4.805 | 1.55E-06 | 3.15E-05 |
| Prevotella_9 | Control | 329.228 | -5.724 | 0.808 | -7.084 | 1.40E-12 | 1.34E-10 |
| Akkermansia | Control | 85.053 | -5.015 | 0.628 | -7.980 | 1.47E-15 | 2.60E-13 |
| Staphylococcus | GDM | 43.140 | 5.135 | 0.743 | 6.916 | 4.66E-12 | 3.91E-10 |
| Ruminococcus | GDM | 442.754 | 5.376 | 0.705 | 7.630 | 2.34E-14 | 2.99E-12 |
| Prevotella_9 | Control | 186.912 | -4.077 | 0.835 | -4.881 | 1.06E-06 | 2.26E-05 |
| Bifidobacterium | GDM | 329.281 | 4.907 | 0.571 | 8.587 | 8.93E-18 | 2.13E-15 |
| [Ruminococcus] gnavus group | GDM | 327.667 | 2.127 | 0.499 | 4.261 | 2.03E-05 | 0.000295 |
| Dorea | GDM | 321.386 | 2.505 | 0.439 | 5.710 | 1.13E-08 | 4.13E-07 |
| Megamonas | GDM | 74.509 | 3.734 | 0.756 | 4.939 | 7.85E-07 | 1.76E-05 |
| Ruminococcus | GDM | 337.333 | 4.267 | 0.732 | 5.829 | 5.58E-09 | 2.23E-07 |
| Bacteroides | GDM | 70.684 | 6.613 | 0.872 | 7.586 | 3.30E-14 | 4.10E-12 |
| Clostridium sensu stricto 1 | GDM | 20.105 | 4.785 | 0.799 | 5.991 | 2.08E-09 | 9.30E-08 |
| Comamonas | Control | 44.825 | -7.296 | 0.672 | -10.852 | 1.94E-27 | 2.20E-24 |
| Erysipelotrichaceae UCG-003 | GDM | 191.702 | 2.695 | 0.673 | 4.005 | 6.21E-05 | 0.00075968 |
| [Ruminococcus] torques group | GDM | 196.263 | 3.051 | 0.659 | 4.628 | 3.69E-06 | 6.67E-05 |
| Bacteroides | Control | 93.860 | -3.851 | 0.778 | -4.951 | 7.40E-07 | 1.67E-05 |
| Anaerostipes | GDM | 321.316 | 2.714 | 0.580 | 4.683 | 2.83E-06 | 5.31E-05 |
| Bifidobacterium | GDM | 26.982 | 4.504 | 0.813 | 5.540 | 3.03E-08 | 9.84E-07 |
| Prevotella_7 | GDM | 13.982 | 4.252 | 0.837 | 5.078 | 3.81E-07 | 9.25E-06 |
| Parabacteroides | GDM | 72.649 | 4.659 | 0.772 | 6.038 | 1.56E-09 | 7.22E-08 |
| Stenotrophomonas | Control | 2.421 | -2.600 | 0.408 | -6.372 | 1.86E-10 | 1.11E-08 |
| Lachnospiraceae ND3007 group | GDM | 184.667 | 2.801 | 0.567 | 4.940 | 7.80E-07 | 1.75E-05 |
| Bacteroides | Control | 54.930 | -7.593 | 0.666 | -11.399 | 4.21E-30 | 6.36E-27 |
| Bacteroides | Control | 195.351 | -3.876 | 0.787 | -4.924 | 8.47E-07 | 1.86E-05 |
| NA | Control | 51.368 | -6.667 | 0.671 | -9.933 | 2.98E-23 | 1.80E-20 |
| [Eubacterium] hallii group | GDM | 176.632 | 4.335 | 0.520 | 8.341 | 7.40E-17 | 1.52E-14 |
| Ligilactobacillus | GDM | 86.807 | 6.385 | 0.810 | 7.884 | 3.17E-15 | 4.86E-13 |
| [Eubacterium] hallii group | GDM | 178.175 | 3.404 | 0.568 | 5.992 | 2.07E-09 | 9.27E-08 |
| Streptococcus | GDM | 12.105 | 2.554 | 0.634 | 4.028 | 5.63E-05 | 0.00069548 |
| Akkermansia | GDM | 30.842 | 5.409 | 0.818 | 6.614 | 3.75E-11 | 2.57E-09 |
| Cutibacterium | GDM | 20.965 | 4.846 | 0.806 | 6.011 | 1.84E-09 | 8.39E-08 |
| Prevotella_9 | Control | 93.193 | -8.364 | 0.619 | -13.502 | 1.52E-41 | 1.38E-37 |
| Collinsella | GDM | 13.316 | 4.180 | 0.781 | 5.351 | 8.77E-08 | 2.45E-06 |
| Haemophilus | Control | 25.579 | -3.264 | 0.650 | -5.018 | 5.21E-07 | 1.22E-05 |
| Intestinibacter | GDM | 99.947 | 4.670 | 0.526 | 8.884 | 6.42E-19 | 2.01E-16 |
| Megamonas | GDM | 27.246 | 5.228 | 0.851 | 6.143 | 8.11E-10 | 4.06E-08 |
| NA | Control | 48.421 | -5.908 | 0.677 | -8.725 | 2.66E-18 | 7.52E-16 |
| Bacteroides | Control | 107.772 | -6.878 | 0.666 | -10.323 | 5.53E-25 | 4.17E-22 |
| Fusobacterium | GDM | 12.351 | 3.979 | 0.734 | 5.418 | 6.04E-08 | 1.81E-06 |
| Collinsella | GDM | 29.298 | 5.334 | 0.858 | 6.216 | 5.11E-10 | 2.68E-08 |
| Klebsiella | Control | 2.158 | -2.358 | 0.489 | -4.822 | 1.42E-06 | 2.93E-05 |
| Veillonella | Control | 21.632 | -3.761 | 0.666 | -5.645 | 1.66E-08 | 5.86E-07 |
| UCG-005 | Control | 123.579 | -4.145 | 0.758 | -5.471 | 4.48E-08 | 1.40E-06 |
| NA | Control | 13.474 | -3.295 | 0.573 | -5.751 | 8.87E-09 | 3.32E-07 |
| Staphylococcus | GDM | 35.053 | 5.595 | 0.840 | 6.660 | 2.74E-11 | 1.93E-09 |
| Ruminococcus | GDM | 80.474 | 6.801 | 0.743 | 9.149 | 5.73E-20 | 2.36E-17 |
| Bacteroides | GDM | 23.526 | 5.014 | 0.825 | 6.079 | 1.21E-09 | 5.79E-08 |
| Dorea | GDM | 89.825 | 2.363 | 0.442 | 5.348 | 8.89E-08 | 2.48E-06 |
| Alistipes | Control | 33.491 | -5.017 | 0.686 | -7.310 | 2.67E-13 | 2.78E-11 |
| UCG-002 | GDM | 81.351 | 2.936 | 0.697 | 4.211 | 2.54E-05 | 0.00035555 |
| Klebsiella | Control | 2.702 | -2.820 | 0.515 | -5.480 | 4.24E-08 | 1.33E-06 |
| Lelliottia | Control | 10.649 | -3.424 | 0.610 | -5.613 | 1.99E-08 | 6.81E-07 |
| Bacillus | GDM | 4.526 | 2.561 | 0.652 | 3.926 | 8.65E-05 | 0.00099704 |
| Prevotella_9 | Control | 50.825 | -7.480 | 0.651 | -11.482 | 1.62E-30 | 2.94E-27 |
| Ruminococcus | GDM | 31.140 | 5.423 | 0.845 | 6.420 | 1.37E-10 | 8.30E-09 |
| Prevotella_9 | GDM | 14.912 | 4.346 | 0.800 | 5.434 | 5.51E-08 | 1.66E-06 |
| Veillonella | Control | 6.930 | -4.016 | 0.575 | -6.986 | 2.83E-12 | 2.56E-10 |
| Prevotella_9 | Control | 99.211 | -5.620 | 0.745 | -7.543 | 4.60E-14 | 5.48E-12 |
| Blautia | GDM | 100.649 | 4.786 | 0.635 | 7.534 | 4.91E-14 | 5.78E-12 |
| Prevotella_9 | Control | 58.754 | -4.457 | 0.732 | -6.092 | 1.12E-09 | 5.41E-08 |
| Lactobacillus | GDM | 6.175 | 3.035 | 0.702 | 4.324 | 1.53E-05 | 0.0002312 |
| Oscillibacter | Control | 95.860 | -1.887 | 0.445 | -4.238 | 2.26E-05 | 0.00032443 |
| Blautia | GDM | 89.772 | 4.085 | 0.586 | 6.976 | 3.03E-12 | 2.72E-10 |
| Escherichia-Shigella | Control | 2.175 | -2.375 | 0.490 | -4.849 | 1.24E-06 | 2.60E-05 |
| Prevotella_9 | Control | 42.421 | -7.215 | 0.617 | -11.689 | 1.45E-31 | 4.39E-28 |
| NA | GDM | 57.123 | 6.304 | 0.830 | 7.598 | 3.00E-14 | 3.78E-12 |
| Roseburia | GDM | 42.632 | 3.828 | 0.653 | 5.864 | 4.52E-09 | 1.89E-07 |
| Sutterella | Control | 62.298 | -5.002 | 0.742 | -6.737 | 1.62E-11 | 1.20E-09 |
| Klebsiella | GDM | 5.509 | 2.862 | 0.709 | 4.038 | 5.39E-05 | 0.00067567 |
| Bacteroides | Control | 104.421 | -5.019 | 0.808 | -6.214 | 5.17E-10 | 2.69E-08 |
| Prevotella_9 | Control | 10.667 | -5.147 | 0.549 | -9.375 | 6.94E-21 | 3.44E-18 |
| Sellimonas | GDM | 89.140 | 5.657 | 0.711 | 7.956 | 1.78E-15 | 2.98E-13 |
| Lelliottia | Control | 1.772 | -1.907 | 0.459 | -4.156 | 3.24E-05 | 0.00043606 |
| Desulfovibrio | Control | 1.842 | -2.000 | 0.398 | -5.030 | 4.90E-07 | 1.16E-05 |
| Sellimonas | GDM | 78.193 | 3.988 | 0.630 | 6.330 | 2.44E-10 | 1.40E-08 |
| Megasphaera | Control | 45.088 | -7.304 | 0.628 | -11.636 | 2.69E-31 | 6.10E-28 |
| Bacteroides | GDM | 15.877 | 4.438 | 0.792 | 5.601 | 2.13E-08 | 7.17E-07 |
| UCG-002 | Control | 24.842 | -5.645 | 0.605 | -9.327 | 1.09E-20 | 4.71E-18 |
| Prevotella_9 | Control | 9.930 | -3.191 | 0.663 | -4.813 | 1.49E-06 | 3.03E-05 |
| [Eubacterium] hallii group | GDM | 72.719 | 3.662 | 0.579 | 6.322 | 2.58E-10 | 1.46E-08 |
| Erysipelatoclostridium | GDM | 53.614 | 4.078 | 0.650 | 6.274 | 3.52E-10 | 1.92E-08 |
| Anaerostipes | GDM | 67.491 | 3.391 | 0.635 | 5.340 | 9.32E-08 | 2.57E-06 |
| Prevotella_9 | GDM | 14.158 | 4.270 | 0.784 | 5.445 | 5.17E-08 | 1.58E-06 |
| Bifidobacterium | GDM | 9.807 | 3.727 | 0.723 | 5.154 | 2.55E-07 | 6.41E-06 |
| Sphingomonas | Control | 27.439 | -6.573 | 0.618 | -10.638 | 1.98E-26 | 1.63E-23 |
| Prevotella_9 | GDM | 14.333 | 4.288 | 0.798 | 5.375 | 7.67E-08 | 2.21E-06 |
| Bacteroides | Control | 12.316 | -5.369 | 0.602 | -8.916 | 4.82E-19 | 1.75E-16 |
| Prevotella_9 | Control | 47.842 | -5.097 | 0.712 | -7.160 | 8.06E-13 | 8.21E-11 |
| Klebsiella | Control | 2.912 | -2.966 | 0.504 | -5.889 | 3.89E-09 | 1.67E-07 |
| Clostridium sensu stricto 1 | GDM | 10.070 | 3.767 | 0.736 | 5.120 | 3.05E-07 | 7.60E-06 |
| Enterococcus | GDM | 25.070 | 5.107 | 0.691 | 7.391 | 1.46E-13 | 1.56E-11 |
| Veillonella | Control | 24.263 | -4.323 | 0.622 | -6.952 | 3.60E-12 | 3.11E-10 |
| Escherichia-Shigella | Control | 3.596 | -3.358 | 0.527 | -6.368 | 1.92E-10 | 1.13E-08 |
| Ligilactobacillus | GDM | 21.526 | 3.199 | 0.752 | 4.253 | 2.11E-05 | 0.00030533 |
| Prevotella_9 | GDM | 14.263 | 4.281 | 0.786 | 5.447 | 5.13E-08 | 1.57E-06 |
| Catenibacterium | GDM | 5.211 | 2.777 | 0.701 | 3.960 | 7.49E-05 | 0.00088675 |
| Ruminococcus | GDM | 59.947 | 3.170 | 0.782 | 4.051 | 5.10E-05 | 0.00064328 |
| Lactobacillus | Control | 31.351 | -5.754 | 0.661 | -8.703 | 3.24E-18 | 8.90E-16 |
| Prevotella_9 | Control | 50.070 | -5.320 | 0.703 | -7.570 | 3.75E-14 | 4.52E-12 |
| Prevotella_9 | Control | 5.000 | -3.931 | 0.557 | -7.058 | 1.69E-12 | 1.58E-10 |
| Megamonas | GDM | 9.632 | 3.700 | 0.786 | 4.706 | 2.53E-06 | 4.85E-05 |
| Bacteroides | GDM | 22.386 | 3.749 | 0.721 | 5.201 | 1.99E-07 | 5.08E-06 |
| Prevotella_9 | Control | 5.895 | -4.205 | 0.569 | -7.394 | 1.42E-13 | 1.55E-11 |
| Faecalibacterium | Control | 28.404 | -3.174 | 0.742 | -4.279 | 1.88E-05 | 0.00027559 |
| Bradyrhizobium | Control | 24.860 | -5.364 | 0.651 | -8.242 | 1.69E-16 | 3.26E-14 |
| Megasphaera | GDM | 94.351 | 5.239 | 0.871 | 6.018 | 1.76E-09 | 8.11E-08 |
| Barnesiella | Control | 7.789 | -2.646 | 0.622 | -4.252 | 2.12E-05 | 0.00030575 |
| Streptococcus | GDM | 36.491 | 5.125 | 0.759 | 6.751 | 1.47E-11 | 1.11E-09 |
| Prevotella_9 | Control | 3.439 | -3.276 | 0.540 | -6.065 | 1.32E-09 | 6.19E-08 |
| Veillonella | GDM | 10.228 | 3.790 | 0.745 | 5.087 | 3.64E-07 | 8.91E-06 |
| Enterobacter | Control | 16.526 | -2.658 | 0.592 | -4.489 | 7.15E-06 | 0.00011825 |
| NA | Control | 16.719 | -3.755 | 0.654 | -5.741 | 9.41E-09 | 3.50E-07 |
| Megamonas | GDM | 6.579 | 3.130 | 0.743 | 4.210 | 2.55E-05 | 0.00035655 |
| Cutibacterium | GDM | 11.298 | 3.937 | 0.713 | 5.521 | 3.37E-08 | 1.08E-06 |
| Prevotella_9 | GDM | 11.807 | 4.002 | 0.766 | 5.224 | 1.75E-07 | 4.56E-06 |
| Bacteroides | Control | 5.316 | -4.033 | 0.579 | -6.964 | 3.32E-12 | 2.95E-10 |
| Prevotella | Control | 8.246 | -4.745 | 0.534 | -8.892 | 6.02E-19 | 2.01E-16 |
| [Eubacterium] hallii group | GDM | 50.123 | 4.576 | 0.683 | 6.698 | 2.11E-11 | 1.52E-09 |
| Prevotella_9 | Control | 12.281 | -5.364 | 0.603 | -8.896 | 5.81E-19 | 2.01E-16 |
| Bacteroides | Control | 7.509 | -4.596 | 0.587 | -7.826 | 5.02E-15 | 7.34E-13 |
| Bacteroides | Control | 6.456 | -2.702 | 0.626 | -4.318 | 1.57E-05 | 0.00023695 |
| Blautia | GDM | 57.281 | 3.885 | 0.698 | 5.569 | 2.56E-08 | 8.43E-07 |
| Blautia | GDM | 51.351 | 6.150 | 0.703 | 8.754 | 2.07E-18 | 6.04E-16 |
| NA | Control | 8.614 | -2.393 | 0.601 | -3.985 | 6.75E-05 | 0.00081118 |
| Bacteroides | Control | 10.298 | -2.900 | 0.675 | -4.299 | 1.71E-05 | 0.00025482 |
| Paraprevotella | GDM | 12.667 | 4.106 | 0.774 | 5.306 | 1.12E-07 | 3.05E-06 |
| Roseburia | GDM | 38.035 | 2.868 | 0.632 | 4.536 | 5.73E-06 | 9.74E-05 |
| CAG-352 | GDM | 9.807 | 3.727 | 0.813 | 4.585 | 4.53E-06 | 7.98E-05 |
| Salmonella | Control | 2.263 | -2.459 | 0.438 | -5.614 | 1.98E-08 | 6.80E-07 |
| Bacteroides | GDM | 12.842 | 4.126 | 0.784 | 5.264 | 1.41E-07 | 3.74E-06 |
| Bacteroides | Control | 22.140 | -6.254 | 0.578 | -10.815 | 2.91E-27 | 2.93E-24 |
| Faecalibacterium | Control | 10.754 | -3.009 | 0.675 | -4.457 | 8.30E-06 | 0.00013529 |
| Veillonella | Control | 4.123 | -3.600 | 0.523 | -6.878 | 6.09E-12 | 4.93E-10 |
| Prevotella_9 | Control | 34.509 | -3.781 | 0.719 | -5.260 | 1.44E-07 | 3.81E-06 |
| Bacteroides | Control | 15.509 | -5.418 | 0.578 | -9.370 | 7.22E-21 | 3.44E-18 |
| Ruminococcus | GDM | 30.702 | 5.402 | 0.839 | 6.436 | 1.22E-10 | 7.60E-09 |
| Faecalibacterium | GDM | 7.947 | 3.414 | 0.743 | 4.594 | 4.35E-06 | 7.69E-05 |
| Bacteroides | Control | 6.877 | -3.826 | 0.583 | -6.559 | 5.42E-11 | 3.64E-09 |
| Streptococcus | GDM | 30.632 | 5.399 | 0.843 | 6.406 | 1.49E-10 | 8.95E-09 |
| Bacteroides | Control | 9.281 | -4.931 | 0.584 | -8.450 | 2.92E-17 | 6.45E-15 |
| Barnesiella | GDM | 23.018 | 3.964 | 0.758 | 5.231 | 1.69E-07 | 4.41E-06 |
| Ralstonia | Control | 8.175 | -4.116 | 0.557 | -7.391 | 1.45E-13 | 1.56E-11 |
| Enterobacter | Control | 1.807 | -1.954 | 0.462 | -4.227 | 2.37E-05 | 0.00033798 |
| Prevotella | Control | 14.105 | -5.576 | 0.571 | -9.759 | 1.69E-22 | 9.58E-20 |
| Finegoldia | GDM | 7.509 | 3.070 | 0.671 | 4.575 | 4.76E-06 | 8.27E-05 |
| Bacteroides | Control | 10.018 | -5.050 | 0.590 | -8.556 | 1.17E-17 | 2.71E-15 |
| Bacteroides | GDM | 5.579 | 2.881 | 0.711 | 4.052 | 5.09E-05 | 0.00064283 |
| Barnesiella | Control | 15.298 | -5.699 | 0.624 | -9.138 | 6.37E-20 | 2.51E-17 |
| Bacteroides | Control | 22.632 | -6.287 | 0.582 | -10.796 | 3.58E-27 | 3.24E-24 |
| Collinsella | GDM | 6.596 | 3.134 | 0.721 | 4.345 | 1.39E-05 | 0.00021374 |
| Bacteroides | Control | 3.544 | -3.331 | 0.543 | -6.133 | 8.65E-10 | 4.28E-08 |
| Bacteroides | Control | 3.982 | -3.414 | 0.551 | -6.195 | 5.82E-10 | 3.01E-08 |
| Agathobacter | GDM | 18.351 | 3.186 | 0.784 | 4.063 | 4.85E-05 | 0.00061766 |
| Prevotella_9 | Control | 17.211 | -3.968 | 0.665 | -5.969 | 2.39E-09 | 1.06E-07 |
| Nitrospira | Control | 18.053 | -5.948 | 0.594 | -10.017 | 1.28E-23 | 8.31E-21 |
| Gardnerella | GDM | 8.474 | 3.510 | 0.772 | 4.548 | 5.41E-06 | 9.30E-05 |
| Bacteroides | Control | 6.474 | -4.358 | 0.534 | -8.160 | 3.35E-16 | 6.33E-14 |
| Blautia | GDM | 32.544 | 4.779 | 0.694 | 6.890 | 5.58E-12 | 4.64E-10 |
| Ligilactobacillus | Control | 23.421 | -3.914 | 0.668 | -5.855 | 4.77E-09 | 1.96E-07 |
| Sutterella | Control | 9.737 | -5.006 | 0.594 | -8.431 | 3.43E-17 | 7.39E-15 |
| Prevotella_9 | GDM | 7.860 | 3.397 | 0.737 | 4.609 | 4.04E-06 | 7.23E-05 |
| Prevotella_9 | Control | 5.053 | -3.948 | 0.556 | -7.104 | 1.21E-12 | 1.20E-10 |
| CAG-352 | GDM | 6.596 | 3.134 | 0.754 | 4.156 | 3.24E-05 | 0.00043606 |
| Sutterella | Control | 45.158 | -2.697 | 0.682 | -3.953 | 7.71E-05 | 0.00090899 |
| NA | Control | 7.281 | -4.547 | 0.535 | -8.503 | 1.84E-17 | 4.17E-15 |
| Ruminococcus | GDM | 26.018 | 3.425 | 0.724 | 4.733 | 2.21E-06 | 4.32E-05 |
| Bacteroides | GDM | 5.596 | 2.886 | 0.722 | 3.999 | 6.37E-05 | 0.00077686 |
| Pediococcus | Control | 1.737 | -1.858 | 0.455 | -4.084 | 4.42E-05 | 0.0005712 |
| Ruminococcus | GDM | 9.912 | 3.743 | 0.772 | 4.850 | 1.23E-06 | 2.60E-05 |
| Eggerthella | GDM | 27.930 | 4.868 | 0.607 | 8.024 | 1.02E-15 | 1.86E-13 |
| Bacteroides | Control | 3.088 | -3.077 | 0.529 | -5.817 | 5.99E-09 | 2.35E-07 |
| NA | GDM | 31.825 | 3.158 | 0.682 | 4.628 | 3.69E-06 | 6.67E-05 |
| Megamonas | Control | 5.596 | -3.188 | 0.573 | -5.561 | 2.68E-08 | 8.77E-07 |
| Prevotella_9 | Control | 9.509 | -3.744 | 0.591 | -6.334 | 2.39E-10 | 1.38E-08 |
| Odoribacter | Control | 17.596 | -2.505 | 0.600 | -4.174 | 2.99E-05 | 0.00041103 |
| Weissella | GDM | 26.789 | 5.204 | 0.603 | 8.628 | 6.27E-18 | 1.53E-15 |
| NA | Control | 8.105 | -3.549 | 0.582 | -6.101 | 1.05E-09 | 5.16E-08 |
| Prevotella_9 | Control | 5.421 | -4.066 | 0.531 | -7.653 | 1.96E-14 | 2.53E-12 |
| Odoribacter | Control | 30.123 | -2.921 | 0.585 | -4.991 | 6.00E-07 | 1.40E-05 |
| NA | Control | 5.737 | -4.160 | 0.540 | -7.706 | 1.30E-14 | 1.74E-12 |
| Clostridium sensu stricto 1 | GDM | 7.333 | 3.293 | 0.706 | 4.665 | 3.09E-06 | 5.74E-05 |
| Coprobacter | Control | 8.404 | -4.364 | 0.586 | -7.451 | 9.27E-14 | 1.05E-11 |
| Ruminococcus | GDM | 8.947 | 3.591 | 0.748 | 4.800 | 1.58E-06 | 3.20E-05 |
| NA | GDM | 5.965 | 2.982 | 0.713 | 4.183 | 2.88E-05 | 0.00039547 |
| Faecalicoccus | GDM | 8.140 | 3.450 | 0.697 | 4.952 | 7.35E-07 | 1.66E-05 |
| NA | Control | 12.263 | -5.362 | 0.575 | -9.328 | 1.08E-20 | 4.71E-18 |
| Blautia | GDM | 33.561 | 5.532 | 0.840 | 6.584 | 4.58E-11 | 3.12E-09 |
| Pseudomonas | Control | 3.649 | -3.134 | 0.481 | -6.512 | 7.40E-11 | 4.69E-09 |
| Butyrivibrio | Control | 8.123 | -3.921 | 0.550 | -7.134 | 9.75E-13 | 9.82E-11 |
| Bacteroides | GDM | 6.070 | 3.009 | 0.723 | 4.161 | 3.16E-05 | 0.00042927 |
| Megamonas | GDM | 9.825 | 3.730 | 0.715 | 5.215 | 1.84E-07 | 4.78E-06 |
| Corynebacterium | GDM | 9.123 | 3.620 | 0.731 | 4.950 | 7.41E-07 | 1.67E-05 |
| Nitrospira | GDM | 16.386 | 4.485 | 0.797 | 5.625 | 1.85E-08 | 6.45E-07 |
| Erysipelatoclostridium | GDM | 10.088 | 2.930 | 0.604 | 4.852 | 1.22E-06 | 2.58E-05 |
| Bacillus | Control | 14.105 | -5.576 | 0.582 | -9.580 | 9.67E-22 | 5.15E-19 |
| Lachnospiraceae FCS020 group | GDM | 27.754 | 3.438 | 0.609 | 5.644 | 1.66E-08 | 5.86E-07 |
| Romboutsia | GDM | 6.439 | 3.098 | 0.700 | 4.427 | 9.57E-06 | 0.00015379 |
| Parabacteroides | GDM | 8.877 | 3.403 | 0.720 | 4.730 | 2.25E-06 | 4.37E-05 |
| Marvinbryantia | GDM | 24.789 | 3.215 | 0.605 | 5.310 | 1.10E-07 | 3.00E-06 |
| Collinsella | GDM | 13.561 | 4.207 | 0.727 | 5.783 | 7.34E-09 | 2.81E-07 |
| Clostridium sensu stricto 1 | GDM | 33.404 | 3.135 | 0.703 | 4.460 | 8.18E-06 | 0.0001336 |
| Blautia | GDM | 8.614 | 3.534 | 0.763 | 4.633 | 3.60E-06 | 6.54E-05 |
| Anaerostipes | GDM | 11.035 | 3.902 | 0.701 | 5.563 | 2.65E-08 | 8.72E-07 |
| Dorea | GDM | 12.579 | 4.096 | 0.699 | 5.857 | 4.71E-09 | 1.96E-07 |
| Parabacteroides | GDM | 6.614 | 3.138 | 0.711 | 4.414 | 1.01E-05 | 0.00016225 |
| Lachnospira | Control | 4.368 | -3.700 | 0.562 | -6.579 | 4.74E-11 | 3.20E-09 |
| Parabacteroides | Control | 19.509 | -5.168 | 0.596 | -8.672 | 4.23E-18 | 1.10E-15 |
| Bacteroides | Control | 7.123 | -4.512 | 0.583 | -7.740 | 9.95E-15 | 1.37E-12 |
| NA | Control | 6.737 | -4.200 | 0.554 | -7.575 | 3.58E-14 | 4.39E-12 |
| Bacteroides | Control | 4.526 | -3.275 | 0.530 | -6.183 | 6.27E-10 | 3.21E-08 |
| Bacteroides | GDM | 9.175 | 3.453 | 0.712 | 4.848 | 1.24E-06 | 2.60E-05 |
| NA | Control | 4.386 | -3.707 | 0.527 | -7.039 | 1.93E-12 | 1.79E-10 |
| Alistipes | Control | 3.158 | -2.077 | 0.528 | -3.936 | 8.28E-05 | 0.0009624 |
| Prevotella | Control | 8.211 | -4.738 | 0.545 | -8.693 | 3.53E-18 | 9.42E-16 |
| Bacteroides | Control | 3.456 | -3.285 | 0.541 | -6.077 | 1.23E-09 | 5.85E-08 |
| Dialister | Control | 2.298 | -2.492 | 0.496 | -5.027 | 4.98E-07 | 1.18E-05 |
| Desulfovibrio | GDM | 4.842 | 2.665 | 0.670 | 3.976 | 7.00E-05 | 0.00083742 |
| Prevotella_9 | Control | 3.193 | -2.839 | 0.514 | -5.519 | 3.41E-08 | 1.09E-06 |
| Bacteroides | GDM | 7.930 | 3.411 | 0.727 | 4.692 | 2.71E-06 | 5.09E-05 |
| Blautia | GDM | 20.719 | 4.829 | 0.739 | 6.536 | 6.30E-11 | 4.08E-09 |
| Lachnoclostridium | GDM | 16.982 | 2.752 | 0.651 | 4.230 | 2.34E-05 | 0.00033463 |
| Dialister | GDM | 7.211 | 3.268 | 0.746 | 4.380 | 1.19E-05 | 0.00018567 |
| Klebsiella | Control | 3.140 | -3.109 | 0.494 | -6.288 | 3.22E-10 | 1.79E-08 |
| Alloprevotella | GDM | 6.246 | 3.052 | 0.722 | 4.227 | 2.37E-05 | 0.00033798 |
| UCG-003 | Control | 4.807 | -2.510 | 0.539 | -4.658 | 3.19E-06 | 5.91E-05 |
| Bacteroides | GDM | 5.982 | 2.987 | 0.710 | 4.205 | 2.61E-05 | 0.00036498 |
| NA | Control | 6.035 | -4.127 | 0.521 | -7.923 | 2.32E-15 | 3.76E-13 |
| Parabacteroides | Control | 14.982 | -3.105 | 0.652 | -4.759 | 1.95E-06 | 3.86E-05 |
| Parabacteroides | Control | 17.439 | -5.896 | 0.581 | -10.148 | 3.40E-24 | 2.37E-21 |
| Dubosiella | Control | 2.772 | -2.870 | 0.482 | -5.958 | 2.56E-09 | 1.13E-07 |
| Turicibacter | GDM | 10.719 | 2.598 | 0.638 | 4.074 | 4.63E-05 | 0.0005948 |
| NA | Control | 5.737 | -4.160 | 0.538 | -7.736 | 1.03E-14 | 1.39E-12 |
| Prevotella_9 | Control | 2.509 | -2.434 | 0.481 | -5.055 | 4.30E-07 | 1.04E-05 |
| Klebsiella | Control | 3.877 | -2.716 | 0.553 | -4.915 | 8.87E-07 | 1.94E-05 |
| NA | Control | 2.860 | -2.931 | 0.502 | -5.833 | 5.45E-09 | 2.19E-07 |
| Limosilactobacillus | GDM | 7.333 | 3.293 | 0.707 | 4.660 | 3.17E-06 | 5.87E-05 |
| Anaerostipes | GDM | 21.070 | 4.258 | 0.727 | 5.854 | 4.81E-09 | 1.96E-07 |
| Barnesiella | Control | 3.439 | -3.276 | 0.504 | -6.496 | 8.27E-11 | 5.17E-09 |
| Blautia | GDM | 8.667 | 2.934 | 0.669 | 4.385 | 1.16E-05 | 0.00018208 |
| [Ruminococcus] torques group | GDM | 7.368 | 2.890 | 0.707 | 4.089 | 4.32E-05 | 0.00056383 |
| NA | Control | 2.175 | -2.375 | 0.471 | -5.045 | 4.53E-07 | 1.08E-05 |
| Blautia | GDM | 5.316 | 2.807 | 0.715 | 3.926 | 8.64E-05 | 0.00099704 |
| Ruminococcus | Control | 5.439 | -3.272 | 0.571 | -5.734 | 9.82E-09 | 3.63E-07 |
| NA | Control | 3.526 | -3.322 | 0.529 | -6.285 | 3.27E-10 | 1.80E-08 |
| Erysipelatoclostridium | GDM | 18.456 | 2.937 | 0.575 | 5.108 | 3.26E-07 | 8.07E-06 |
| Parabacteroides | GDM | 6.386 | 3.085 | 0.686 | 4.496 | 6.91E-06 | 0.00011539 |
| Atopobium | GDM | 5.561 | 2.876 | 0.721 | 3.991 | 6.59E-05 | 0.00079818 |
| Tyzzerella | GDM | 12.281 | 2.506 | 0.572 | 4.378 | 1.20E-05 | 0.00018702 |
| NA | Control | 8.193 | -3.766 | 0.551 | -6.840 | 7.93E-12 | 6.20E-10 |
| Blautia | GDM | 6.596 | 3.134 | 0.693 | 4.522 | 6.14E-06 | 0.00010379 |
| Romboutsia | GDM | 5.158 | 2.761 | 0.701 | 3.939 | 8.19E-05 | 0.0009532 |
| Faecalibacterium | Control | 3.825 | -3.468 | 0.532 | -6.520 | 7.05E-11 | 4.50E-09 |
| NA | Control | 5.807 | -3.685 | 0.542 | -6.803 | 1.02E-11 | 7.85E-10 |
| Escherichia-Shigella | Control | 3.228 | -3.160 | 0.499 | -6.329 | 2.47E-10 | 1.41E-08 |
| UCG-003 | Control | 15.982 | -3.106 | 0.586 | -5.301 | 1.15E-07 | 3.12E-06 |
| Eubacterium | GDM | 13.930 | 4.246 | 0.667 | 6.370 | 1.89E-10 | 1.12E-08 |
| Lachnoclostridium | GDM | 13.825 | 4.235 | 0.715 | 5.926 | 3.11E-09 | 1.35E-07 |
| Bacteroides | GDM | 13.439 | 3.259 | 0.680 | 4.791 | 1.66E-06 | 3.33E-05 |
| Lachnoclostridium | GDM | 16.807 | 3.594 | 0.704 | 5.102 | 3.37E-07 | 8.27E-06 |
| Barnesiella | GDM | 7.246 | 3.275 | 0.695 | 4.713 | 2.44E-06 | 4.70E-05 |
| Olsenella | GDM | 6.895 | 3.201 | 0.708 | 4.520 | 6.19E-06 | 0.00010442 |
| Faecalibacterium | Control | 2.667 | -2.794 | 0.494 | -5.653 | 1.57E-08 | 5.59E-07 |
| Bradyrhizobium | Control | 6.842 | -4.447 | 0.539 | -8.257 | 1.49E-16 | 3.01E-14 |
| Butyricimonas | Control | 23.526 | -2.837 | 0.647 | -4.384 | 1.17E-05 | 0.00018277 |
| Klebsiella | Control | 5.772 | -4.170 | 0.532 | -7.833 | 4.77E-15 | 7.09E-13 |
| Bacteroides | Control | 5.561 | -4.109 | 0.551 | -7.461 | 8.57E-14 | 9.96E-12 |
| Atopobium | GDM | 5.719 | 2.919 | 0.724 | 4.029 | 5.60E-05 | 0.00069305 |
| Prevotella | Control | 3.772 | -3.443 | 0.515 | -6.688 | 2.26E-11 | 1.61E-09 |
| Megasphaera | GDM | 7.842 | 3.394 | 0.684 | 4.959 | 7.09E-07 | 1.61E-05 |
| Bacteroides | GDM | 8.228 | 3.466 | 0.738 | 4.697 | 2.64E-06 | 5.00E-05 |
| Pseudarthrobacter | Control | 4.421 | -3.721 | 0.527 | -7.058 | 1.69E-12 | 1.58E-10 |
| Gardnerella | GDM | 5.877 | 2.960 | 0.717 | 4.128 | 3.66E-05 | 0.00048717 |
| Acinetobacter | Control | 3.333 | -2.135 | 0.514 | -4.156 | 3.23E-05 | 0.00043606 |
| NA | GDM | 12.035 | 3.627 | 0.641 | 5.658 | 1.54E-08 | 5.48E-07 |
| Bacteroides | Control | 12.684 | -3.161 | 0.643 | -4.918 | 8.72E-07 | 1.91E-05 |
| Prevotella | Control | 2.439 | -2.615 | 0.484 | -5.405 | 6.49E-08 | 1.93E-06 |
| Bacteroides | Control | 2.105 | -1.893 | 0.467 | -4.056 | 5.00E-05 | 0.00063383 |
| Parvimonas | GDM | 6.053 | 3.004 | 0.720 | 4.170 | 3.04E-05 | 0.00041613 |
| Anaerostipes | GDM | 9.544 | 3.687 | 0.744 | 4.955 | 7.25E-07 | 1.64E-05 |
| CAG-352 | GDM | 13.439 | 4.193 | 0.722 | 5.809 | 6.30E-09 | 2.45E-07 |
| NA | GDM | 15.491 | 3.472 | 0.635 | 5.467 | 4.58E-08 | 1.43E-06 |
| Clostridium sensu stricto 1 | GDM | 8.719 | 3.552 | 0.734 | 4.842 | 1.28E-06 | 2.65E-05 |
| NA | Control | 2.877 | -2.631 | 0.499 | -5.269 | 1.37E-07 | 3.66E-06 |
| Prevotella | Control | 2.281 | -2.476 | 0.476 | -5.201 | 1.98E-07 | 5.08E-06 |
| Prevotella_9 | Control | 3.421 | -3.267 | 0.523 | -6.242 | 4.33E-10 | 2.31E-08 |
| Sutterella | Control | 21.263 | -2.806 | 0.710 | -3.951 | 7.79E-05 | 0.00091288 |
| Parabacteroides | Control | 2.596 | -2.741 | 0.475 | -5.773 | 7.78E-09 | 2.95E-07 |
| NA | GDM | 6.316 | 3.069 | 0.691 | 4.444 | 8.84E-06 | 0.00014327 |
| Parabacteroides | Control | 2.807 | -2.848 | 0.481 | -5.915 | 3.31E-09 | 1.44E-07 |
| Alloprevotella | Control | 2.333 | -2.524 | 0.478 | -5.276 | 1.32E-07 | 3.54E-06 |
| Bacteroides | Control | 1.982 | -2.170 | 0.478 | -4.540 | 5.63E-06 | 9.59E-05 |
| Blautia | GDM | 15.474 | 4.401 | 0.757 | 5.813 | 6.15E-09 | 2.40E-07 |
| Lachnospira | Control | 2.140 | -2.340 | 0.488 | -4.795 | 1.62E-06 | 3.26E-05 |
| Senegalimassilia | GDM | 5.807 | 2.680 | 0.680 | 3.941 | 8.13E-05 | 0.00094708 |
| Bacteroides | Control | 1.877 | -2.044 | 0.469 | -4.359 | 1.30E-05 | 0.00020103 |
| Parabacteroides | Control | 5.561 | -3.953 | 0.534 | -7.406 | 1.30E-13 | 1.44E-11 |
| Bacteroides | GDM | 9.667 | 3.706 | 0.736 | 5.036 | 4.75E-07 | 1.13E-05 |
| NA | GDM | 7.316 | 3.290 | 0.672 | 4.894 | 9.90E-07 | 2.14E-05 |
| Sutterella | Control | 14.193 | -4.250 | 0.613 | -6.938 | 3.97E-12 | 3.40E-10 |
| Blautia | GDM | 19.281 | 4.723 | 0.815 | 5.793 | 6.92E-09 | 2.66E-07 |
| Lactobacillus | Control | 2.175 | -2.375 | 0.490 | -4.849 | 1.24E-06 | 2.60E-05 |
| Prevotella | Control | 4.263 | -2.751 | 0.559 | -4.919 | 8.69E-07 | 1.91E-05 |
| [Eubacterium] brachy group | GDM | 11.228 | 3.928 | 0.552 | 7.114 | 1.13E-12 | 1.12E-10 |
| Lachnoclostridium | Control | 2.754 | -2.858 | 0.499 | -5.730 | 1.00E-08 | 3.69E-07 |
| Sutterella | Control | 16.754 | -3.106 | 0.670 | -4.636 | 3.55E-06 | 6.47E-05 |
| Barnesiella | Control | 4.544 | -3.768 | 0.550 | -6.853 | 7.26E-12 | 5.77E-10 |
| Olsenella | GDM | 6.474 | 3.106 | 0.678 | 4.582 | 4.62E-06 | 8.08E-05 |
| Collinsella | GDM | 7.632 | 3.353 | 0.687 | 4.880 | 1.06E-06 | 2.26E-05 |
| Prevotella | Control | 4.298 | -2.919 | 0.551 | -5.298 | 1.17E-07 | 3.15E-06 |
| Phascolarctobacterium | Control | 9.088 | -3.232 | 0.596 | -5.422 | 5.88E-08 | 1.77E-06 |
| Oscillibacter | Control | 13.158 | -2.263 | 0.515 | -4.394 | 1.11E-05 | 0.0001765 |
| Parabacteroides | GDM | 5.579 | 2.881 | 0.694 | 4.153 | 3.29E-05 | 0.00044201 |
| Desulfovibrio | Control | 2.930 | -2.753 | 0.446 | -6.175 | 6.63E-10 | 3.36E-08 |
| Bacteroides | Control | 1.772 | -1.907 | 0.459 | -4.156 | 3.24E-05 | 0.00043606 |
| NA | Control | 5.351 | -4.044 | 0.513 | -7.884 | 3.16E-15 | 4.86E-13 |
| Bradyrhizobium | Control | 2.737 | -2.845 | 0.487 | -5.848 | 4.99E-09 | 2.03E-07 |
| Sutterella | GDM | 6.439 | 3.098 | 0.679 | 4.559 | 5.14E-06 | 8.92E-05 |
| Lactobacillus | Control | 5.070 | -3.954 | 0.489 | -8.088 | 6.05E-16 | 1.12E-13 |
| Sellimonas | GDM | 6.439 | 3.098 | 0.692 | 4.480 | 7.47E-06 | 0.00012265 |
| Dubosiella | Control | 2.088 | -2.285 | 0.450 | -5.079 | 3.80E-07 | 9.25E-06 |
| Parabacteroides | Control | 2.386 | -2.570 | 0.464 | -5.536 | 3.09E-08 | 9.98E-07 |
| Bacteroides | Control | 2.456 | -2.629 | 0.486 | -5.413 | 6.21E-08 | 1.86E-06 |
| Rhodoplanes | Control | 2.877 | -2.943 | 0.476 | -6.180 | 6.40E-10 | 3.26E-08 |
| Faecalibacterium | Control | 1.754 | -1.883 | 0.457 | -4.120 | 3.78E-05 | 0.0005013 |
| Prevotella_9 | Control | 4.491 | -3.748 | 0.546 | -6.864 | 6.70E-12 | 5.37E-10 |
| Blautia | GDM | 10.158 | 3.779 | 0.692 | 5.458 | 4.82E-08 | 1.48E-06 |
| Sellimonas | GDM | 7.158 | 3.257 | 0.722 | 4.508 | 6.53E-06 | 0.00010919 |
| [Ruminococcus] torques group | GDM | 11.649 | 3.148 | 0.670 | 4.700 | 2.60E-06 | 4.96E-05 |
| Dubosiella | Control | 2.088 | -2.285 | 0.485 | -4.712 | 2.45E-06 | 4.71E-05 |
| Bacteroides | GDM | 4.860 | 2.670 | 0.661 | 4.041 | 5.33E-05 | 0.0006691 |
| NA | Control | 3.965 | -3.531 | 0.518 | -6.817 | 9.31E-12 | 7.21E-10 |
| Mitsuokella | Control | 6.895 | -3.514 | 0.571 | -6.155 | 7.49E-10 | 3.77E-08 |
| UCG-002 | Control | 2.614 | -2.755 | 0.492 | -5.599 | 2.16E-08 | 7.24E-07 |
| Sutterella | Control | 3.561 | -3.340 | 0.531 | -6.288 | 3.22E-10 | 1.79E-08 |
| NA | Control | 1.912 | -2.087 | 0.453 | -4.609 | 4.06E-06 | 7.25E-05 |
| UCG-002 | GDM | 6.632 | 3.142 | 0.714 | 4.398 | 1.09E-05 | 0.00017347 |
| Bacteroides | Control | 4.053 | -2.589 | 0.521 | -4.971 | 6.65E-07 | 1.53E-05 |
| Lapillicoccus | Control | 3.526 | -3.322 | 0.507 | -6.555 | 5.57E-11 | 3.68E-09 |
| NA | Control | 7.491 | -3.497 | 0.582 | -6.013 | 1.82E-09 | 8.34E-08 |
| NA | GDM | 8.105 | 3.443 | 0.722 | 4.769 | 1.85E-06 | 3.67E-05 |
| Shuttleworthia | GDM | 6.158 | 3.030 | 0.645 | 4.701 | 2.58E-06 | 4.94E-05 |
| Megasphaera | GDM | 9.491 | 3.272 | 0.685 | 4.774 | 1.80E-06 | 3.60E-05 |
| Butyricicoccus | GDM | 5.947 | 2.978 | 0.706 | 4.220 | 2.45E-05 | 0.00034554 |
| Kitasatospora | Control | 1.754 | -1.883 | 0.439 | -4.287 | 1.81E-05 | 0.00026736 |
| Ruminococcus | GDM | 11.140 | 3.916 | 0.753 | 5.201 | 1.99E-07 | 5.08E-06 |
| Blautia | GDM | 8.211 | 3.463 | 0.737 | 4.699 | 2.61E-06 | 4.96E-05 |
| NA | Control | 3.825 | -3.468 | 0.504 | -6.883 | 5.86E-12 | 4.83E-10 |
| Phascolarctobacterium | Control | 3.333 | -3.219 | 0.501 | -6.425 | 1.32E-10 | 8.08E-09 |
| Acidaminococcus | Control | 2.684 | -2.807 | 0.514 | -5.463 | 4.67E-08 | 1.45E-06 |
| UCG-005 | GDM | 5.965 | 2.982 | 0.711 | 4.197 | 2.71E-05 | 0.0003773 |
| Blautia | GDM | 10.211 | 3.787 | 0.742 | 5.107 | 3.27E-07 | 8.07E-06 |
| Faecalibacterium | Control | 2.596 | -2.741 | 0.492 | -5.571 | 2.53E-08 | 8.37E-07 |
| Clostridium sensu stricto 1 | GDM | 5.719 | 2.919 | 0.718 | 4.064 | 4.83E-05 | 0.00061568 |
| Parabacteroides | GDM | 8.035 | 3.022 | 0.688 | 4.391 | 1.13E-05 | 0.00017817 |
| Sellimonas | GDM | 10.544 | 3.109 | 0.660 | 4.711 | 2.46E-06 | 4.73E-05 |
| Clostridium sensu stricto 1 | GDM | 6.737 | 3.166 | 0.709 | 4.465 | 8.01E-06 | 0.00013106 |
| Blastococcus | Control | 1.982 | -2.170 | 0.459 | -4.730 | 2.24E-06 | 4.36E-05 |
| Oscillospira | Control | 9.877 | -3.658 | 0.582 | -6.286 | 3.27E-10 | 1.80E-08 |
| NA | Control | 3.368 | -3.238 | 0.497 | -6.520 | 7.05E-11 | 4.50E-09 |
| Desulfovibrio | GDM | 8.772 | 3.561 | 0.729 | 4.883 | 1.05E-06 | 2.25E-05 |
| Geodermatophilus | Control | 2.632 | -2.768 | 0.478 | -5.795 | 6.84E-09 | 2.64E-07 |
| Bradyrhizobium | Control | 3.684 | -3.401 | 0.512 | -6.644 | 3.06E-11 | 2.12E-09 |
| Pedomicrobium | Control | 2.509 | -2.672 | 0.474 | -5.638 | 1.72E-08 | 6.05E-07 |
| Gaiella | Control | 2.772 | -2.870 | 0.484 | -5.931 | 3.00E-09 | 1.31E-07 |
| Shuttleworthia | GDM | 9.123 | 3.620 | 0.663 | 5.459 | 4.80E-08 | 1.48E-06 |
| Acidothermus | Control | 3.702 | -3.409 | 0.520 | -6.556 | 5.51E-11 | 3.67E-09 |
| Sutterella | Control | 3.193 | -3.140 | 0.532 | -5.896 | 3.72E-09 | 1.60E-07 |
| NA | Control | 4.333 | -2.262 | 0.543 | -4.165 | 3.11E-05 | 0.00042445 |
| Bryobacter | Control | 4.035 | -3.562 | 0.524 | -6.797 | 1.07E-11 | 8.15E-10 |
| Bacteroides | Control | 9.789 | -4.281 | 0.558 | -7.668 | 1.74E-14 | 2.29E-12 |
| Streptomyces | Control | 3.456 | -3.285 | 0.506 | -6.497 | 8.21E-11 | 5.16E-09 |
| Butyricicoccus | GDM | 11.228 | 3.928 | 0.707 | 5.559 | 2.71E-08 | 8.83E-07 |
| Pseudonocardia | Control | 2.526 | -2.686 | 0.488 | -5.502 | 3.75E-08 | 1.19E-06 |
| UCG-003 | Control | 7.947 | -3.645 | 0.533 | -6.841 | 7.87E-12 | 6.20E-10 |
| Acidothermus | Control | 5.386 | -4.055 | 0.519 | -7.811 | 5.66E-15 | 8.10E-13 |
| Ruminococcus | Control | 4.860 | -3.883 | 0.527 | -7.371 | 1.69E-13 | 1.79E-11 |
| [Eubacterium] hallii group | GDM | 6.439 | 3.098 | 0.711 | 4.359 | 1.30E-05 | 0.00020103 |
| Lachnospiraceae UCG-010 | Control | 6.825 | -3.729 | 0.532 | -7.014 | 2.32E-12 | 2.12E-10 |
| Prevotella_9 | Control | 3.193 | -3.140 | 0.515 | -6.099 | 1.07E-09 | 5.20E-08 |
| Megasphaera | GDM | 4.912 | 2.687 | 0.663 | 4.053 | 5.05E-05 | 0.00063916 |
| NA | Control | 3.579 | -3.349 | 0.482 | -6.952 | 3.60E-12 | 3.11E-10 |
| NA | Control | 1.877 | -2.044 | 0.469 | -4.359 | 1.30E-05 | 0.00020103 |
| Mitsuokella | Control | 1.982 | -2.170 | 0.460 | -4.719 | 2.37E-06 | 4.59E-05 |
| Blautia | GDM | 5.053 | 2.730 | 0.630 | 4.335 | 1.45E-05 | 0.00022183 |
| Sutterella | Control | 3.018 | -3.033 | 0.526 | -5.762 | 8.34E-09 | 3.15E-07 |
| Crossiella | Control | 5.982 | -4.229 | 0.530 | -7.972 | 1.56E-15 | 2.71E-13 |
| NA | Control | 2.316 | -2.508 | 0.463 | -5.412 | 6.23E-08 | 1.86E-06 |
| Veillonella | Control | 1.930 | -2.109 | 0.454 | -4.639 | 3.49E-06 | 6.40E-05 |
| NA | Control | 3.737 | -3.426 | 0.512 | -6.688 | 2.27E-11 | 1.61E-09 |
| NA | GDM | 8.105 | 2.597 | 0.654 | 3.973 | 7.09E-05 | 0.00084601 |
| Acidothermus | Control | 2.105 | -2.304 | 0.467 | -4.936 | 7.99E-07 | 1.77E-05 |
| Pseudonocardia | Control | 2.000 | -2.190 | 0.461 | -4.754 | 1.99E-06 | 3.93E-05 |
| Afipia | Control | 2.667 | -2.794 | 0.478 | -5.841 | 5.18E-09 | 2.09E-07 |
| NA | Control | 2.105 | -2.304 | 0.456 | -5.055 | 4.30E-07 | 1.04E-05 |
| Butyricicoccus | GDM | 8.719 | 3.552 | 0.690 | 5.151 | 2.58E-07 | 6.49E-06 |
| Lactococcus | GDM | 5.456 | 2.847 | 0.655 | 4.344 | 1.40E-05 | 0.00021491 |
| Bacteroides | Control | 2.404 | -2.585 | 0.482 | -5.369 | 7.94E-08 | 2.28E-06 |
| Oscillibacter | Control | 8.439 | -2.671 | 0.579 | -4.613 | 3.98E-06 | 7.15E-05 |
| NA | Control | 2.140 | -2.340 | 0.469 | -4.991 | 6.02E-07 | 1.40E-05 |
| Vibrio | Control | 1.544 | -1.555 | 0.392 | -3.970 | 7.19E-05 | 0.00085509 |
| Alistipes | Control | 3.561 | -3.340 | 0.525 | -6.367 | 1.93E-10 | 1.13E-08 |
| Ottowia | GDM | 4.737 | 2.631 | 0.659 | 3.993 | 6.53E-05 | 0.00079278 |
| Blautia | GDM | 5.018 | 2.719 | 0.668 | 4.074 | 4.63E-05 | 0.0005948 |
| Bacteroides | Control | 2.035 | -2.015 | 0.439 | -4.592 | 4.38E-06 | 7.73E-05 |
| Clostridium sensu stricto 1 | Control | 2.088 | -2.285 | 0.450 | -5.079 | 3.80E-07 | 9.25E-06 |
| Subdoligranulum | GDM | 5.105 | 2.746 | 0.680 | 4.041 | 5.33E-05 | 0.0006691 |
| Pseudolabrys | Control | 2.667 | -2.794 | 0.479 | -5.829 | 5.56E-09 | 2.23E-07 |
| NA | Control | 2.842 | -2.919 | 0.487 | -5.997 | 2.01E-09 | 9.04E-08 |
| Enterobacter | Control | 3.053 | -3.055 | 0.509 | -6.003 | 1.93E-09 | 8.76E-08 |
| NA | Control | 2.456 | -2.629 | 0.468 | -5.619 | 1.92E-08 | 6.67E-07 |
| Lachnoclostridium | GDM | 8.842 | 3.573 | 0.732 | 4.882 | 1.05E-06 | 2.26E-05 |
| Streptomyces | Control | 2.140 | -2.340 | 0.472 | -4.960 | 7.04E-07 | 1.60E-05 |
| NA | GDM | 7.947 | 2.680 | 0.659 | 4.066 | 4.79E-05 | 0.00061174 |
| NA | Control | 6.684 | -3.136 | 0.569 | -5.507 | 3.65E-08 | 1.17E-06 |
| NA | GDM | 5.351 | 2.637 | 0.647 | 4.076 | 4.58E-05 | 0.0005914 |
| Pseudarthrobacter | Control | 2.684 | -2.807 | 0.479 | -5.855 | 4.77E-09 | 1.96E-07 |
| NA | Control | 2.421 | -2.600 | 0.485 | -5.355 | 8.54E-08 | 2.40E-06 |
| Eggerthella | GDM | 4.754 | 2.637 | 0.665 | 3.963 | 7.39E-05 | 0.00087679 |
| Prevotella | Control | 1.877 | -2.044 | 0.435 | -4.695 | 2.67E-06 | 5.04E-05 |
| Sphingomonas | Control | 4.123 | -2.177 | 0.551 | -3.952 | 7.76E-05 | 0.00091203 |
| Conexibacter | Control | 1.807 | -1.954 | 0.443 | -4.410 | 1.03E-05 | 0.00016494 |
| NA | Control | 3.526 | -3.322 | 0.533 | -6.237 | 4.47E-10 | 2.37E-08 |
| Coprobacter | Control | 2.351 | -2.539 | 0.479 | -5.300 | 1.16E-07 | 3.12E-06 |
| Oribacterium | Control | 5.316 | -4.033 | 0.518 | -7.782 | 7.11E-15 | 9.91E-13 |
| Prevotella | Control | 6.807 | -4.439 | 0.538 | -8.249 | 1.60E-16 | 3.14E-14 |
| NA | GDM | 8.228 | 3.466 | 0.723 | 4.797 | 1.61E-06 | 3.25E-05 |
| Prevotellaceae UCG-001 | Control | 8.965 | -4.877 | 0.536 | -9.095 | 9.50E-20 | 3.59E-17 |
| NA | Control | 3.298 | -3.200 | 0.526 | -6.087 | 1.15E-09 | 5.55E-08 |
| NA | Control | 3.088 | -3.077 | 0.494 | -6.225 | 4.83E-10 | 2.54E-08 |
| Sphingomonas | Control | 2.439 | -2.615 | 0.486 | -5.379 | 7.49E-08 | 2.17E-06 |
| Mitsuokella | GDM | 4.912 | 2.687 | 0.611 | 4.394 | 1.11E-05 | 0.00017632 |
| NA | Control | 2.526 | -2.686 | 0.473 | -5.676 | 1.38E-08 | 4.94E-07 |
| Collinsella | GDM | 4.667 | 2.608 | 0.638 | 4.086 | 4.38E-05 | 0.00057062 |
| Kribbella | Control | 1.930 | -2.109 | 0.439 | -4.803 | 1.56E-06 | 3.18E-05 |
| Conexibacter | Control | 1.912 | -2.087 | 0.455 | -4.585 | 4.55E-06 | 7.99E-05 |
| Sellimonas | GDM | 6.333 | 3.073 | 0.684 | 4.494 | 7.00E-06 | 0.00011656 |
| Fournierella | GDM | 5.281 | 2.797 | 0.652 | 4.289 | 1.79E-05 | 0.0002646 |
| Alistipes | Control | 2.018 | -2.209 | 0.481 | -4.598 | 4.26E-06 | 7.56E-05 |
| Bacillus | Control | 1.719 | -1.833 | 0.453 | -4.047 | 5.18E-05 | 0.00065249 |
| Parabacteroides | Control | 2.105 | -2.304 | 0.486 | -4.740 | 2.14E-06 | 4.20E-05 |
| Dubosiella | Control | 1.667 | -1.755 | 0.446 | -3.934 | 8.35E-05 | 0.00096679 |
| NA | Control | 2.140 | -2.340 | 0.488 | -4.795 | 1.62E-06 | 3.26E-05 |
| NA | Control | 2.632 | -2.768 | 0.476 | -5.820 | 5.90E-09 | 2.32E-07 |
| NA | Control | 5.754 | -4.165 | 0.527 | -7.903 | 2.72E-15 | 4.32E-13 |
| Parabacteroides | Control | 3.140 | -2.685 | 0.498 | -5.391 | 7.00E-08 | 2.05E-06 |
| Sphingomonas | Control | 1.719 | -1.833 | 0.435 | -4.217 | 2.47E-05 | 0.00034796 |
| [Ruminococcus] torques group | GDM | 6.860 | 2.577 | 0.653 | 3.947 | 7.92E-05 | 0.00092547 |
| NA | GDM | 4.702 | 2.620 | 0.659 | 3.976 | 7.01E-05 | 0.00083742 |
| Adlercreutzia | GDM | 6.316 | 3.069 | 0.677 | 4.535 | 5.77E-06 | 9.80E-05 |
| Candidatus Udaeobacter | Control | 2.298 | -2.492 | 0.465 | -5.362 | 8.22E-08 | 2.34E-06 |
| NA | Control | 1.754 | -1.883 | 0.438 | -4.302 | 1.69E-05 | 0.00025295 |
| Steroidobacter | Control | 2.105 | -2.304 | 0.467 | -4.936 | 7.98E-07 | 1.77E-05 |
| Rugosimonospora | Control | 1.737 | -1.858 | 0.438 | -4.246 | 2.17E-05 | 0.00031277 |
| NA | Control | 3.684 | -3.401 | 0.489 | -6.955 | 3.53E-12 | 3.10E-10 |
| Coprobacter | Control | 2.316 | -2.508 | 0.478 | -5.252 | 1.51E-07 | 3.98E-06 |
| NA | Control | 2.175 | -2.375 | 0.459 | -5.178 | 2.24E-07 | 5.67E-06 |
| Lachnospiraceae NK4A136 group | Control | 3.456 | -3.285 | 0.488 | -6.729 | 1.70E-11 | 1.24E-09 |
| NA | Control | 2.105 | -2.304 | 0.469 | -4.913 | 8.98E-07 | 1.96E-05 |
| NA | GDM | 5.105 | 2.746 | 0.671 | 4.095 | 4.22E-05 | 0.00055107 |
| Butyricimonas | Control | 2.965 | -3.000 | 0.489 | -6.139 | 8.30E-10 | 4.13E-08 |
| NA | GDM | 5.035 | 2.725 | 0.664 | 4.104 | 4.07E-05 | 0.00053193 |
| NA | Control | 3.386 | -3.248 | 0.483 | -6.731 | 1.69E-11 | 1.23E-09 |
| [Eubacterium] fissicatena group | GDM | 5.263 | 2.792 | 0.588 | 4.745 | 2.08E-06 | 4.11E-05 |
| NA | Control | 1.702 | -1.807 | 0.451 | -4.010 | 6.07E-05 | 0.00074348 |
| Acidaminococcus | Control | 2.579 | -2.728 | 0.509 | -5.357 | 8.45E-08 | 2.39E-06 |
| NA | Control | 2.789 | -2.789 | 0.474 | -5.879 | 4.13E-09 | 1.76E-07 |
| NA | Control | 2.491 | -2.658 | 0.473 | -5.617 | 1.94E-08 | 6.72E-07 |
| NA | Control | 2.053 | -2.248 | 0.466 | -4.821 | 1.43E-06 | 2.94E-05 |
| NA | Control | 2.474 | -2.644 | 0.504 | -5.245 | 1.57E-07 | 4.11E-06 |
| NA | Control | 1.614 | -1.672 | 0.406 | -4.123 | 3.74E-05 | 0.00049747 |
| NA | Control | 4.439 | -3.728 | 0.500 | -7.455 | 9.00E-14 | 1.03E-11 |
| Allobaculum | Control | 2.246 | -2.443 | 0.446 | -5.481 | 4.22E-08 | 1.33E-06 |
| NA | Control | 2.035 | -2.229 | 0.455 | -4.894 | 9.87E-07 | 2.13E-05 |
| Dactylosporangium | Control | 2.281 | -2.476 | 0.462 | -5.360 | 8.31E-08 | 2.35E-06 |
| NA | Control | 2.035 | -2.229 | 0.449 | -4.968 | 6.77E-07 | 1.55E-05 |
| Microvirga | Control | 4.158 | -2.358 | 0.541 | -4.354 | 1.34E-05 | 0.00020564 |
| Coprococcus | Control | 2.053 | -2.248 | 0.433 | -5.196 | 2.03E-07 | 5.17E-06 |
| Acidothermus | Control | 1.754 | -1.883 | 0.438 | -4.293 | 1.76E-05 | 0.0002605 |
| Mesorhizobium | Control | 1.737 | -1.858 | 0.455 | -4.084 | 4.42E-05 | 0.0005712 |
| Skermanella | Control | 1.719 | -1.833 | 0.434 | -4.220 | 2.44E-05 | 0.00034496 |
| NA | Control | 1.860 | -2.022 | 0.451 | -4.482 | 7.40E-06 | 0.00012175 |
| NA | Control | 2.982 | -3.011 | 0.478 | -6.297 | 3.03E-10 | 1.70E-08 |
| NA | Control | 2.053 | -2.248 | 0.453 | -4.962 | 6.97E-07 | 1.59E-05 |
| Candidatus Udaeobacter | Control | 1.702 | -1.807 | 0.434 | -4.162 | 3.15E-05 | 0.00042885 |
| Parabacteroides | Control | 2.263 | -2.459 | 0.458 | -5.368 | 7.98E-08 | 2.28E-06 |
| Actinomycetospora | Control | 2.000 | -2.190 | 0.444 | -4.931 | 8.18E-07 | 1.81E-05 |
| NA | Control | 1.930 | -2.109 | 0.458 | -4.599 | 4.25E-06 | 7.55E-05 |
| Microvirga | Control | 1.702 | -1.807 | 0.439 | -4.119 | 3.81E-05 | 0.00050347 |
| NA | Control | 5.158 | -2.561 | 0.546 | -4.695 | 2.67E-06 | 5.04E-05 |
| Lachnospiraceae NK4A136 group | Control | 1.807 | -1.954 | 0.429 | -4.556 | 5.21E-06 | 8.99E-05 |
| NA | Control | 1.947 | -2.129 | 0.442 | -4.814 | 1.48E-06 | 3.03E-05 |
| Reyranella | Control | 1.860 | -2.022 | 0.451 | -4.482 | 7.40E-06 | 0.00012175 |
| Hyphomicrobium | Control | 1.965 | -2.150 | 0.448 | -4.801 | 1.58E-06 | 3.20E-05 |
| Quadrisphaera | Control | 1.860 | -2.022 | 0.467 | -4.327 | 1.51E-05 | 0.00022845 |
| Faecalibacterium | Control | 2.632 | -2.768 | 0.494 | -5.601 | 2.13E-08 | 7.17E-07 |
| NA | Control | 2.316 | -2.508 | 0.460 | -5.447 | 5.11E-08 | 1.57E-06 |
| NA | Control | 2.649 | -2.781 | 0.480 | -5.796 | 6.78E-09 | 2.63E-07 |
| Dongia | Control | 2.649 | -2.781 | 0.465 | -5.977 | 2.27E-09 | 1.01E-07 |
| Burkholderia-Caballeronia-Paraburkholderia | Control | 2.947 | -2.989 | 0.465 | -6.434 | 1.24E-10 | 7.66E-09 |
| NA | Control | 2.789 | -2.883 | 0.499 | -5.777 | 7.62E-09 | 2.90E-07 |
| Blautia | GDM | 6.263 | 3.056 | 0.664 | 4.604 | 4.14E-06 | 7.39E-05 |
| Bacillus | Control | 2.175 | -1.923 | 0.474 | -4.056 | 4.98E-05 | 0.0006324 |
| NA | Control | 2.246 | -2.443 | 0.476 | -5.134 | 2.84E-07 | 7.08E-06 |
| Sphingomonas | Control | 1.737 | -1.858 | 0.455 | -4.084 | 4.42E-05 | 0.0005712 |
| NA | Control | 2.053 | -2.248 | 0.449 | -5.012 | 5.40E-07 | 1.26E-05 |
| NA | Control | 1.965 | -2.150 | 0.458 | -4.692 | 2.71E-06 | 5.09E-05 |
| NA | Control | 6.088 | -3.962 | 0.498 | -7.964 | 1.67E-15 | 2.86E-13 |
| Colidextribacter | Control | 5.123 | -2.387 | 0.544 | -4.389 | 1.14E-05 | 0.00017991 |
| CAG-56 | GDM | 5.070 | 2.735 | 0.671 | 4.076 | 4.59E-05 | 0.00059151 |
| Reyranella | Control | 1.667 | -1.755 | 0.446 | -3.934 | 8.35E-05 | 0.00096679 |
| Ellin6055 | Control | 1.702 | -1.807 | 0.451 | -4.010 | 6.07E-05 | 0.00074348 |
| Bryobacter | Control | 2.439 | -2.615 | 0.468 | -5.588 | 2.29E-08 | 7.67E-07 |
| Labrys | Control | 2.719 | -2.833 | 0.467 | -6.072 | 1.26E-09 | 5.97E-08 |
| Coprobacter | Control | 1.719 | -1.833 | 0.435 | -4.217 | 2.47E-05 | 0.00034796 |
| NA | Control | 1.965 | -2.150 | 0.444 | -4.845 | 1.26E-06 | 2.63E-05 |
| Sphingomonas | Control | 1.702 | -1.807 | 0.451 | -4.010 | 6.07E-05 | 0.00074348 |
| NA | Control | 2.281 | -2.476 | 0.459 | -5.390 | 7.04E-08 | 2.05E-06 |
| NA | Control | 1.912 | -2.087 | 0.454 | -4.601 | 4.21E-06 | 7.49E-05 |
| Streptomyces | Control | 2.509 | -2.672 | 0.470 | -5.689 | 1.28E-08 | 4.61E-07 |
| NA | Control | 3.667 | -3.392 | 0.511 | -6.644 | 3.05E-11 | 2.12E-09 |
| Ruminococcus | Control | 3.596 | -3.358 | 0.474 | -7.088 | 1.36E-12 | 1.31E-10 |
| Christensenellaceae R-7 group | Control | 5.667 | -4.140 | 0.527 | -7.855 | 4.01E-15 | 6.06E-13 |
| NA | Control | 1.772 | -1.907 | 0.441 | -4.329 | 1.50E-05 | 0.00022748 |
| NA | Control | 1.684 | -1.781 | 0.448 | -3.972 | 7.12E-05 | 0.00084857 |
| NA | Control | 2.544 | -2.700 | 0.460 | -5.873 | 4.27E-09 | 1.82E-07 |
| Bacteroides | Control | 2.088 | -2.285 | 0.466 | -4.900 | 9.60E-07 | 2.08E-05 |
| Jatrophihabitans | Control | 2.281 | -2.476 | 0.460 | -5.382 | 7.35E-08 | 2.14E-06 |
| Romboutsia | GDM | 6.175 | 3.035 | 0.703 | 4.317 | 1.58E-05 | 0.00023824 |
| NA | Control | 1.965 | -2.150 | 0.457 | -4.701 | 2.60E-06 | 4.95E-05 |
| NA | Control | 2.228 | -2.426 | 0.492 | -4.928 | 8.32E-07 | 1.84E-05 |
| Streptomyces | Control | 1.965 | -2.150 | 0.461 | -4.668 | 3.04E-06 | 5.67E-05 |
| NA | Control | 1.860 | -2.022 | 0.451 | -4.482 | 7.40E-06 | 0.00012175 |
| NA | Control | 1.737 | -1.858 | 0.455 | -4.084 | 4.42E-05 | 0.0005712 |
| Dialister | Control | 1.947 | -2.129 | 0.456 | -4.670 | 3.01E-06 | 5.62E-05 |
| NA | Control | 2.000 | -2.190 | 0.462 | -4.739 | 2.15E-06 | 4.22E-05 |
| Shuttleworthia | GDM | 4.456 | 2.537 | 0.632 | 4.013 | 6.01E-05 | 0.00073949 |
| NA | Control | 5.281 | -4.022 | 0.515 | -7.810 | 5.72E-15 | 8.10E-13 |
| NA | Control | 1.719 | -1.833 | 0.420 | -4.365 | 1.27E-05 | 0.00019715 |
| NA | Control | 2.754 | -2.858 | 0.467 | -6.114 | 9.72E-10 | 4.79E-08 |
| Negativibacillus | Control | 2.895 | -2.954 | 0.503 | -5.869 | 4.38E-09 | 1.85E-07 |
| Veillonella | Control | 3.842 | -2.155 | 0.478 | -4.511 | 6.46E-06 | 0.00010837 |
| Amycolatopsis | Control | 4.123 | -2.654 | 0.532 | -4.984 | 6.22E-07 | 1.44E-05 |
| NA | Control | 1.825 | -1.977 | 0.464 | -4.261 | 2.04E-05 | 0.00029533 |
| NA | Control | 2.351 | -2.539 | 0.451 | -5.627 | 1.84E-08 | 6.43E-07 |
| NA | GDM | 4.228 | 2.456 | 0.626 | 3.926 | 8.64E-05 | 0.00099704 |
| Psychroglaciecola | Control | 1.825 | -1.977 | 0.430 | -4.594 | 4.35E-06 | 7.69E-05 |
| NA | Control | 2.298 | -2.492 | 0.464 | -5.375 | 7.64E-08 | 2.21E-06 |
| NA | Control | 2.211 | -2.409 | 0.459 | -5.247 | 1.54E-07 | 4.07E-06 |
| Acidibacter | Control | 1.684 | -1.781 | 0.433 | -4.119 | 3.81E-05 | 0.00050347 |
| Christensenellaceae R-7 group | Control | 3.053 | -2.320 | 0.479 | -4.847 | 1.25E-06 | 2.62E-05 |
| Blautia | GDM | 4.684 | 2.614 | 0.651 | 4.013 | 5.99E-05 | 0.00073864 |
| Sphingomonas | Control | 1.860 | -2.022 | 0.433 | -4.665 | 3.08E-06 | 5.73E-05 |
| NA | Control | 2.035 | -2.229 | 0.454 | -4.909 | 9.15E-07 | 1.99E-05 |
| Conexibacter | Control | 2.175 | -2.375 | 0.471 | -5.047 | 4.48E-07 | 1.07E-05 |
| Lachnoclostridium | Control | 5.298 | -2.286 | 0.571 | -4.002 | 6.29E-05 | 0.00076808 |
| Desulfovibrio | Control | 2.474 | -2.221 | 0.488 | -4.556 | 5.22E-06 | 8.99E-05 |
| NA | Control | 1.509 | -1.492 | 0.374 | -3.986 | 6.73E-05 | 0.00080967 |
| NA | Control | 2.368 | -2.555 | 0.445 | -5.747 | 9.09E-09 | 3.39E-07 |
| NA | Control | 3.088 | -2.309 | 0.500 | -4.617 | 3.89E-06 | 7.01E-05 |
| Christensenellaceae R-7 group | Control | 5.035 | -3.823 | 0.515 | -7.430 | 1.09E-13 | 1.22E-11 |
| Kribbella | Control | 1.737 | -1.858 | 0.420 | -4.424 | 9.68E-06 | 0.00015522 |
| Nocardioides | Control | 1.772 | -1.907 | 0.425 | -4.492 | 7.07E-06 | 0.00011726 |
| Butyricicoccus | GDM | 6.246 | 3.052 | 0.679 | 4.491 | 7.08E-06 | 0.00011726 |
| Conexibacter | Control | 2.105 | -2.304 | 0.467 | -4.936 | 7.99E-07 | 1.77E-05 |
| NA | Control | 2.456 | -2.629 | 0.469 | -5.609 | 2.03E-08 | 6.92E-07 |
| Gaiella | Control | 3.000 | -3.022 | 0.475 | -6.358 | 2.04E-10 | 1.19E-08 |
| NA | Control | 1.614 | -1.672 | 0.404 | -4.137 | 3.51E-05 | 0.00046857 |
| Acidicaldus | Control | 1.807 | -1.954 | 0.443 | -4.410 | 1.03E-05 | 0.00016506 |
| Bacillus | Control | 2.018 | -2.209 | 0.463 | -4.768 | 1.86E-06 | 3.70E-05 |
| Lachnospiraceae UCG-001 | Control | 1.737 | -1.858 | 0.443 | -4.192 | 2.76E-05 | 0.00038337 |
| Pseudoduganella | Control | 2.684 | -2.807 | 0.465 | -6.038 | 1.56E-09 | 7.22E-08 |
| Aquamonas | Control | 3.351 | -3.229 | 0.466 | -6.929 | 4.25E-12 | 3.60E-10 |
| Cronobacter | Control | 1.579 | -1.615 | 0.400 | -4.035 | 5.46E-05 | 0.00068219 |
| NA | Control | 3.544 | -2.566 | 0.515 | -4.988 | 6.10E-07 | 1.41E-05 |
| NA | Control | 4.509 | -3.755 | 0.530 | -7.091 | 1.33E-12 | 1.30E-10 |
| Nocardioides | Control | 1.667 | -1.755 | 0.416 | -4.216 | 2.49E-05 | 0.00034934 |
| Nordella | Control | 2.596 | -1.951 | 0.490 | -3.977 | 6.97E-05 | 0.00083557 |
| NA | Control | 2.018 | -2.209 | 0.463 | -4.771 | 1.83E-06 | 3.65E-05 |
| Cloacibacillus | Control | 3.368 | -2.192 | 0.468 | -4.680 | 2.86E-06 | 5.36E-05 |
| Pseudonocardia | Control | 2.035 | -2.229 | 0.463 | -4.814 | 1.48E-06 | 3.03E-05 |
| Asteroleplasma | Control | 1.667 | -1.755 | 0.391 | -4.493 | 7.04E-06 | 0.00011702 |
| Fusicatenibacter | Control | 1.719 | -1.833 | 0.453 | -4.047 | 5.18E-05 | 0.00065249 |
| Quadrisphaera | Control | 1.825 | -1.977 | 0.445 | -4.442 | 8.92E-06 | 0.00014434 |
| NA | Control | 2.281 | -2.476 | 0.459 | -5.391 | 7.00E-08 | 2.05E-06 |
| Marmoricola | Control | 2.439 | -2.615 | 0.467 | -5.603 | 2.11E-08 | 7.17E-07 |
| NA | Control | 2.105 | -2.304 | 0.462 | -4.983 | 6.26E-07 | 1.44E-05 |
| NA | Control | 1.754 | -1.883 | 0.441 | -4.265 | 2.00E-05 | 0.00029175 |
| NA | Control | 1.632 | -1.700 | 0.422 | -4.033 | 5.50E-05 | 0.00068427 |
| Candidatus Solibacter | Control | 1.667 | -1.755 | 0.427 | -4.110 | 3.96E-05 | 0.00051966 |
| Microvirga | Control | 1.912 | -2.087 | 0.442 | -4.724 | 2.31E-06 | 4.48E-05 |
| Mesorhizobium | Control | 2.263 | -2.459 | 0.460 | -5.351 | 8.75E-08 | 2.45E-06 |
| Lachnospira | Control | 2.474 | -2.644 | 0.473 | -5.588 | 2.30E-08 | 7.67E-07 |
| Gemmatimonas | Control | 2.965 | -2.145 | 0.509 | -4.211 | 2.54E-05 | 0.00035555 |
| NA | Control | 2.035 | -2.229 | 0.447 | -4.991 | 6.01E-07 | 1.40E-05 |
| Helicobacter | Control | 2.982 | -3.011 | 0.496 | -6.075 | 1.24E-09 | 5.87E-08 |
| Conexibacter | Control | 1.754 | -1.883 | 0.438 | -4.301 | 1.70E-05 | 0.00025356 |
| Anaerostipes | Control | 2.070 | -2.267 | 0.465 | -4.878 | 1.07E-06 | 2.27E-05 |
| NA | Control | 2.228 | -2.426 | 0.435 | -5.573 | 2.51E-08 | 8.32E-07 |
| Alistipes | Control | 2.719 | -2.833 | 0.497 | -5.702 | 1.18E-08 | 4.31E-07 |
| Gemmatimonas | Control | 2.316 | -2.508 | 0.464 | -5.400 | 6.66E-08 | 1.97E-06 |
| NA | Control | 1.930 | -2.109 | 0.456 | -4.625 | 3.74E-06 | 6.76E-05 |
| NA | Control | 2.053 | -2.248 | 0.436 | -5.155 | 2.53E-07 | 6.39E-06 |
| Blastococcus | Control | 1.614 | -1.672 | 0.419 | -3.990 | 6.62E-05 | 0.00079846 |
| NA | Control | 3.596 | -2.878 | 0.449 | -6.407 | 1.48E-10 | 8.95E-09 |
| NA | GDM | 4.193 | 2.443 | 0.620 | 3.943 | 8.04E-05 | 0.00093905 |
| NA | Control | 1.474 | -1.426 | 0.359 | -3.972 | 7.14E-05 | 0.00084974 |
| NA | Control | 2.316 | -2.508 | 0.461 | -5.439 | 5.36E-08 | 1.63E-06 |
| NA | Control | 3.825 | -3.468 | 0.515 | -6.740 | 1.59E-11 | 1.19E-09 |
| NA | Control | 3.386 | -3.248 | 0.520 | -6.252 | 4.06E-10 | 2.20E-08 |
| NA | Control | 1.895 | -1.843 | 0.433 | -4.252 | 2.12E-05 | 0.00030575 |
| Nitrosospira | Control | 1.632 | -1.700 | 0.422 | -4.034 | 5.49E-05 | 0.00068427 |
| NA | Control | 2.175 | -2.375 | 0.472 | -5.037 | 4.73E-07 | 1.13E-05 |
| Lachnospiraceae NK4A136 group | Control | 2.018 | -2.209 | 0.445 | -4.964 | 6.90E-07 | 1.58E-05 |
| Fictibacillus | Control | 1.807 | -1.954 | 0.445 | -4.387 | 1.15E-05 | 0.00018098 |
| NA | Control | 1.596 | -1.644 | 0.418 | -3.935 | 8.33E-05 | 0.00096679 |
| Candidatus Solibacter | Control | 1.895 | -2.066 | 0.451 | -4.577 | 4.72E-06 | 8.23E-05 |
| NA | Control | 1.895 | -2.066 | 0.436 | -4.734 | 2.20E-06 | 4.30E-05 |
| Rhodopila | Control | 1.667 | -1.755 | 0.414 | -4.235 | 2.29E-05 | 0.00032786 |
| Luedemannella | Control | 2.333 | -2.043 | 0.452 | -4.516 | 6.31E-06 | 0.00010621 |
| NA | Control | 3.825 | -3.468 | 0.515 | -6.734 | 1.65E-11 | 1.21E-09 |
| NA | Control | 1.860 | -2.022 | 0.448 | -4.513 | 6.40E-06 | 0.00010767 |
| NA | Control | 2.105 | -1.843 | 0.440 | -4.187 | 2.83E-05 | 0.00039051 |
| Bacillus | Control | 1.684 | -1.781 | 0.429 | -4.149 | 3.34E-05 | 0.0004471 |
| Allorhizobium-Neorhizobium-Pararhizobium-Rhizobium | Control | 2.035 | -2.229 | 0.437 | -5.106 | 3.29E-07 | 8.11E-06 |
| Steroidobacter | Control | 2.667 | -2.794 | 0.477 | -5.854 | 4.79E-09 | 1.96E-07 |
| Streptomyces | Control | 2.316 | -2.508 | 0.497 | -5.051 | 4.40E-07 | 1.06E-05 |
| NA | Control | 1.684 | -1.781 | 0.414 | -4.303 | 1.68E-05 | 0.00025295 |
| NA | Control | 1.544 | -1.555 | 0.391 | -3.978 | 6.96E-05 | 0.00083493 |
| NA | Control | 2.386 | -2.570 | 0.481 | -5.340 | 9.29E-08 | 2.57E-06 |
| NA | Control | 2.351 | -2.539 | 0.464 | -5.474 | 4.39E-08 | 1.38E-06 |
| Lachnoclostridium | Control | 1.807 | -1.954 | 0.430 | -4.546 | 5.46E-06 | 9.36E-05 |
| Gaiella | Control | 1.754 | -1.883 | 0.423 | -4.456 | 8.36E-06 | 0.00013594 |
| NA | Control | 1.614 | -1.672 | 0.419 | -3.987 | 6.70E-05 | 0.00080719 |
| Bacillus | Control | 1.877 | -2.044 | 0.450 | -4.546 | 5.46E-06 | 9.36E-05 |
| NA | Control | 1.649 | -1.728 | 0.424 | -4.071 | 4.67E-05 | 0.00059911 |
| NA | Control | 2.070 | -2.267 | 0.465 | -4.878 | 1.07E-06 | 2.27E-05 |
| NA | Control | 1.772 | -1.907 | 0.459 | -4.156 | 3.24E-05 | 0.00043606 |
| NA | Control | 1.807 | -1.954 | 0.443 | -4.409 | 1.04E-05 | 0.00016547 |
| NA | Control | 2.649 | -2.781 | 0.478 | -5.823 | 5.77E-09 | 2.28E-07 |
| Rhizobacter | Control | 1.772 | -1.907 | 0.440 | -4.337 | 1.44E-05 | 0.00022022 |
| Lachnospiraceae NK4A136 group | Control | 2.684 | -2.807 | 0.479 | -5.866 | 4.46E-09 | 1.87E-07 |
| Enterorhabdus | Control | 1.702 | -1.807 | 0.417 | -4.335 | 1.46E-05 | 0.00022183 |
| Enterorhabdus | Control | 2.053 | -2.248 | 0.464 | -4.844 | 1.27E-06 | 2.64E-05 |
| Muribaculum | Control | 1.860 | -2.022 | 0.435 | -4.650 | 3.32E-06 | 6.11E-05 |
| NA | Control | 2.211 | -2.409 | 0.456 | -5.279 | 1.30E-07 | 3.50E-06 |
| NA | Control | 1.649 | -1.728 | 0.425 | -4.064 | 4.83E-05 | 0.00061568 |
| NA | Control | 2.053 | -2.248 | 0.447 | -5.025 | 5.05E-07 | 1.19E-05 |
| Lachnospiraceae UCG-001 | Control | 1.789 | -1.931 | 0.442 | -4.371 | 1.24E-05 | 0.00019208 |
| Kinneretia | Control | 1.632 | -1.700 | 0.422 | -4.031 | 5.56E-05 | 0.00068912 |
| Bacillus | Control | 2.070 | -2.267 | 0.465 | -4.878 | 1.07E-06 | 2.27E-05 |
| Dokdonella | Control | 2.298 | -2.492 | 0.462 | -5.399 | 6.70E-08 | 1.98E-06 |
| NA | Control | 1.772 | -1.907 | 0.440 | -4.334 | 1.46E-05 | 0.00022237 |
| Bacteroides | Control | 2.649 | -2.594 | 0.462 | -5.614 | 1.97E-08 | 6.80E-07 |
| NA | Control | 1.719 | -1.833 | 0.434 | -4.226 | 2.38E-05 | 0.00033825 |
| NA | Control | 1.632 | -1.700 | 0.423 | -4.017 | 5.89E-05 | 0.00072757 |
| Ellin6067 | Control | 1.754 | -1.883 | 0.438 | -4.300 | 1.71E-05 | 0.0002546 |
| Enterorhabdus | Control | 1.614 | -1.672 | 0.424 | -3.940 | 8.13E-05 | 0.00094708 |
| NA | Control | 2.421 | -2.600 | 0.482 | -5.391 | 7.01E-08 | 2.05E-06 |
| Bryobacter | Control | 2.228 | -2.426 | 0.457 | -5.314 | 1.07E-07 | 2.94E-06 |
| Mesorhizobium | Control | 1.754 | -1.883 | 0.439 | -4.290 | 1.78E-05 | 0.00026381 |
| Gaiella | Control | 1.789 | -1.931 | 0.442 | -4.373 | 1.23E-05 | 0.00019105 |
| [Ruminococcus] gauvreauii group | Control | 2.175 | -2.375 | 0.454 | -5.235 | 1.65E-07 | 4.31E-06 |
| Anaeromyxobacter | Control | 1.860 | -2.022 | 0.436 | -4.644 | 3.42E-06 | 6.29E-05 |
| NA | Control | 1.772 | -1.907 | 0.445 | -4.285 | 1.83E-05 | 0.00026927 |
| Candidatus Udaeobacter | Control | 1.754 | -1.883 | 0.425 | -4.432 | 9.33E-06 | 0.00015043 |
| Gaiella | Control | 1.842 | -2.000 | 0.434 | -4.612 | 3.99E-06 | 7.16E-05 |
| NA | Control | 3.018 | -3.033 | 0.490 | -6.188 | 6.10E-10 | 3.14E-08 |
| Parabacteroides | Control | 2.263 | -2.308 | 0.443 | -5.206 | 1.93E-07 | 4.97E-06 |
| Oscillibacter | Control | 2.368 | -2.555 | 0.444 | -5.760 | 8.41E-09 | 3.16E-07 |
| Acidothermus | Control | 1.789 | -1.931 | 0.461 | -4.192 | 2.77E-05 | 0.00038337 |
| NA | Control | 1.860 | -2.022 | 0.448 | -4.510 | 6.47E-06 | 0.00010839 |
| Lactobacillus | Control | 2.351 | -2.539 | 0.446 | -5.688 | 1.28E-08 | 4.62E-07 |
| NA | Control | 1.877 | -2.044 | 0.450 | -4.546 | 5.47E-06 | 9.36E-05 |
| Rikenellaceae RC9 gut group | Control | 2.193 | -2.392 | 0.473 | -5.062 | 4.16E-07 | 1.01E-05 |
| NA | Control | 1.702 | -1.807 | 0.451 | -4.010 | 6.07E-05 | 0.00074348 |
| NA | Control | 1.825 | -1.977 | 0.437 | -4.526 | 6.02E-06 | 0.00010195 |
| Nocardioides | Control | 2.000 | -2.190 | 0.444 | -4.933 | 8.08E-07 | 1.79E-05 |
| Lysinibacillus | Control | 1.754 | -1.883 | 0.438 | -4.296 | 1.74E-05 | 0.00025783 |
| Lachnospiraceae NK4A136 group | Control | 1.807 | -1.954 | 0.429 | -4.556 | 5.21E-06 | 8.99E-05 |
| NA | Control | 1.561 | -1.585 | 0.385 | -4.112 | 3.92E-05 | 0.00051567 |
| Enterorhabdus | Control | 2.175 | -2.375 | 0.456 | -5.207 | 1.92E-07 | 4.97E-06 |
| NA | Control | 1.982 | -2.170 | 0.459 | -4.730 | 2.24E-06 | 4.36E-05 |
| NA | Control | 1.930 | -2.109 | 0.454 | -4.640 | 3.48E-06 | 6.39E-05 |
| Ligilactobacillus | Control | 2.281 | -2.476 | 0.422 | -5.871 | 4.33E-09 | 1.83E-07 |
| NA | Control | 1.579 | -1.615 | 0.400 | -4.032 | 5.52E-05 | 0.00068549 |
| NA | Control | 2.842 | -2.605 | 0.471 | -5.536 | 3.09E-08 | 9.98E-07 |
| NA | Control | 1.632 | -1.700 | 0.406 | -4.184 | 2.87E-05 | 0.00039488 |
| Butyricicoccus | Control | 2.193 | -1.991 | 0.472 | -4.222 | 2.42E-05 | 0.00034319 |
| Enterorhabdus | Control | 1.667 | -1.755 | 0.411 | -4.270 | 1.96E-05 | 0.00028676 |
| Amycolatopsis | Control | 1.789 | -1.931 | 0.446 | -4.328 | 1.51E-05 | 0.0002281 |
| Coriobacteriaceae UCG-002 | Control | 2.404 | -2.585 | 0.454 | -5.696 | 1.23E-08 | 4.45E-07 |
| NA | Control | 2.123 | -2.165 | 0.444 | -4.875 | 1.09E-06 | 2.30E-05 |
| UCG-005 | Control | 1.754 | -1.883 | 0.438 | -4.298 | 1.72E-05 | 0.00025573 |
| Nocardioides | Control | 2.263 | -2.459 | 0.459 | -5.362 | 8.23E-08 | 2.34E-06 |
| Prevotella | Control | 1.719 | -1.833 | 0.402 | -4.558 | 5.15E-06 | 8.93E-05 |
| Desulfovibrio | Control | 1.737 | -1.739 | 0.407 | -4.272 | 1.94E-05 | 0.00028459 |
| mle1-7 | Control | 1.596 | -1.644 | 0.416 | -3.952 | 7.74E-05 | 0.00091031 |
| Butyricimonas | Control | 1.825 | -1.977 | 0.451 | -4.388 | 1.14E-05 | 0.00018004 |
| NA | Control | 1.789 | -1.931 | 0.442 | -4.364 | 1.28E-05 | 0.00019782 |
| NA | Control | 1.737 | -1.858 | 0.455 | -4.084 | 4.42E-05 | 0.0005712 |
| Howardella | Control | 2.211 | -2.156 | 0.416 | -5.184 | 2.17E-07 | 5.50E-06 |
| NA | Control | 1.702 | -1.807 | 0.432 | -4.187 | 2.82E-05 | 0.00038995 |
| Bryobacter | Control | 1.561 | -1.585 | 0.397 | -3.992 | 6.54E-05 | 0.00079375 |
| NA | Control | 1.754 | -1.883 | 0.438 | -4.302 | 1.69E-05 | 0.00025301 |
| Anaeromyxobacter | Control | 1.667 | -1.755 | 0.427 | -4.111 | 3.94E-05 | 0.00051718 |
| Rhodoplanes | Control | 1.649 | -1.728 | 0.425 | -4.067 | 4.76E-05 | 0.00060953 |
| Prevotella_9 | Control | 1.825 | -1.977 | 0.431 | -4.583 | 4.57E-06 | 8.02E-05 |
| NA | Control | 1.614 | -1.672 | 0.419 | -3.990 | 6.62E-05 | 0.00079846 |
| Paenarthrobacter | Control | 1.667 | -1.755 | 0.428 | -4.105 | 4.04E-05 | 0.00052956 |
| Odoribacter | Control | 2.123 | -2.322 | 0.452 | -5.137 | 2.79E-07 | 6.98E-06 |
| NA | Control | 1.614 | -1.672 | 0.419 | -3.994 | 6.50E-05 | 0.00079091 |
| Oscillibacter | Control | 1.754 | -1.883 | 0.423 | -4.454 | 8.41E-06 | 0.00013662 |
| Mitsuokella | Control | 1.632 | -1.700 | 0.408 | -4.165 | 3.11E-05 | 0.00042445 |
| NA | Control | 1.702 | -1.807 | 0.434 | -4.162 | 3.15E-05 | 0.00042885 |
| Candidatus Solibacter | Control | 1.789 | -1.931 | 0.461 | -4.192 | 2.77E-05 | 0.00038337 |
| Iamia | Control | 1.737 | -1.858 | 0.436 | -4.260 | 2.05E-05 | 0.00029633 |
| NA | Control | 2.246 | -2.143 | 0.448 | -4.785 | 1.71E-06 | 3.41E-05 |
| NA | Control | 1.947 | -2.129 | 0.440 | -4.843 | 1.28E-06 | 2.65E-05 |
| Gemmatimonas | Control | 1.667 | -1.755 | 0.411 | -4.268 | 1.97E-05 | 0.00028793 |
| Gaiella | Control | 1.877 | -2.044 | 0.450 | -4.545 | 5.49E-06 | 9.37E-05 |
| Enterorhabdus | Control | 1.596 | -1.644 | 0.416 | -3.954 | 7.69E-05 | 0.00090834 |
| NA | Control | 1.614 | -1.672 | 0.419 | -3.990 | 6.62E-05 | 0.00079846 |
| NA | Control | 1.544 | -1.555 | 0.392 | -3.963 | 7.39E-05 | 0.00087679 |
| NA | Control | 1.614 | -1.672 | 0.406 | -4.120 | 3.78E-05 | 0.0005013 |
| NA | Control | 1.965 | -2.150 | 0.428 | -5.021 | 5.14E-07 | 1.21E-05 |
| NA | Control | 2.544 | -2.700 | 0.472 | -5.720 | 1.06E-08 | 3.90E-07 |
| Bacillus | Control | 1.719 | -1.833 | 0.434 | -4.225 | 2.39E-05 | 0.00033988 |
| NA | Control | 2.228 | -2.426 | 0.458 | -5.301 | 1.15E-07 | 3.12E-06 |
| Pedomicrobium | Control | 1.632 | -1.700 | 0.419 | -4.056 | 4.98E-05 | 0.0006324 |
| Butyricicoccus | Control | 2.158 | -2.358 | 0.454 | -5.197 | 2.02E-07 | 5.16E-06 |
| NA | Control | 1.719 | -1.833 | 0.434 | -4.223 | 2.41E-05 | 0.00034229 |
| NA | Control | 1.561 | -1.585 | 0.397 | -3.996 | 6.44E-05 | 0.000784 |
| Mycobacterium | Control | 1.912 | -1.976 | 0.426 | -4.633 | 3.60E-06 | 6.54E-05 |
| NA | Control | 1.632 | -1.700 | 0.408 | -4.172 | 3.02E-05 | 0.00041353 |
| NA | Control | 1.684 | -1.781 | 0.430 | -4.145 | 3.39E-05 | 0.00045328 |
| Lachnospiraceae FCS020 group | Control | 1.754 | -1.883 | 0.425 | -4.431 | 9.36E-06 | 0.00015062 |
| NA | Control | 1.579 | -1.615 | 0.400 | -4.033 | 5.52E-05 | 0.00068549 |
| Colidextribacter | Control | 1.684 | -1.781 | 0.401 | -4.439 | 9.03E-06 | 0.0001459 |
| NA | Control | 2.140 | -2.340 | 0.457 | -5.119 | 3.07E-07 | 7.62E-06 |
| Parvibacter | Control | 1.754 | -1.883 | 0.438 | -4.302 | 1.69E-05 | 0.00025295 |
| Krasilnikovia | Control | 1.684 | -1.781 | 0.429 | -4.149 | 3.34E-05 | 0.0004471 |
| NA | Control | 1.930 | -2.109 | 0.455 | -4.639 | 3.51E-06 | 6.41E-05 |
| NA | Control | 1.860 | -2.022 | 0.435 | -4.652 | 3.29E-06 | 6.08E-05 |
| NA | Control | 2.246 | -2.443 | 0.458 | -5.331 | 9.78E-08 | 2.69E-06 |
| Ramlibacter | Control | 1.702 | -1.807 | 0.432 | -4.187 | 2.82E-05 | 0.00038995 |
| Cellvibrio | Control | 1.667 | -1.755 | 0.427 | -4.111 | 3.94E-05 | 0.00051718 |
| NA | Control | 1.930 | -2.109 | 0.438 | -4.816 | 1.47E-06 | 3.02E-05 |
| Bifidobacterium | Control | 1.772 | -1.907 | 0.436 | -4.378 | 1.20E-05 | 0.00018702 |
| NA | Control | 1.842 | -2.000 | 0.446 | -4.479 | 7.49E-06 | 0.0001227 |
| Conexibacter | Control | 1.596 | -1.644 | 0.416 | -3.954 | 7.70E-05 | 0.00090834 |
| NA | Control | 1.649 | -1.728 | 0.409 | -4.230 | 2.34E-05 | 0.00033463 |
| NA | Control | 1.772 | -1.907 | 0.440 | -4.338 | 1.43E-05 | 0.0002196 |
| NA | Control | 1.632 | -1.700 | 0.409 | -4.162 | 3.16E-05 | 0.00042927 |
| NA | Control | 1.614 | -1.672 | 0.403 | -4.146 | 3.38E-05 | 0.00045185 |
| Bryobacter | Control | 1.596 | -1.644 | 0.416 | -3.951 | 7.80E-05 | 0.00091288 |
| Bryobacter | Control | 1.895 | -2.066 | 0.451 | -4.577 | 4.72E-06 | 8.23E-05 |
| MND1 | Control | 1.632 | -1.700 | 0.422 | -4.033 | 5.50E-05 | 0.00068427 |
| NA | Control | 1.737 | -1.858 | 0.436 | -4.262 | 2.03E-05 | 0.000295 |
| NA | Control | 1.632 | -1.700 | 0.412 | -4.127 | 3.68E-05 | 0.00048986 |
| NA | Control | 1.667 | -1.755 | 0.412 | -4.262 | 2.03E-05 | 0.000295 |
| NA | Control | 1.649 | -1.728 | 0.424 | -4.073 | 4.64E-05 | 0.00059527 |
| NA | Control | 1.596 | -1.644 | 0.407 | -4.036 | 5.43E-05 | 0.00067992 |
| NA | Control | 1.596 | -1.644 | 0.416 | -3.951 | 7.80E-05 | 0.00091288 |
| NA | Control | 1.596 | -1.644 | 0.416 | -3.954 | 7.70E-05 | 0.00090834 |

**Supplementary Fig. S1** Differentially abundant ASVs identified from DeSeq2 based analysis between GDM and non GDM mothers belonging to (**a**) Chinese; (**b**) Malay; and (**c**) Indian ethnic groups. DeSeq2 package v1.38.3 was used to produce the differential AVSs while ggplot2 v3.4.4 was used to generate the figure.

**(b)**

**(c)**

**(a)**


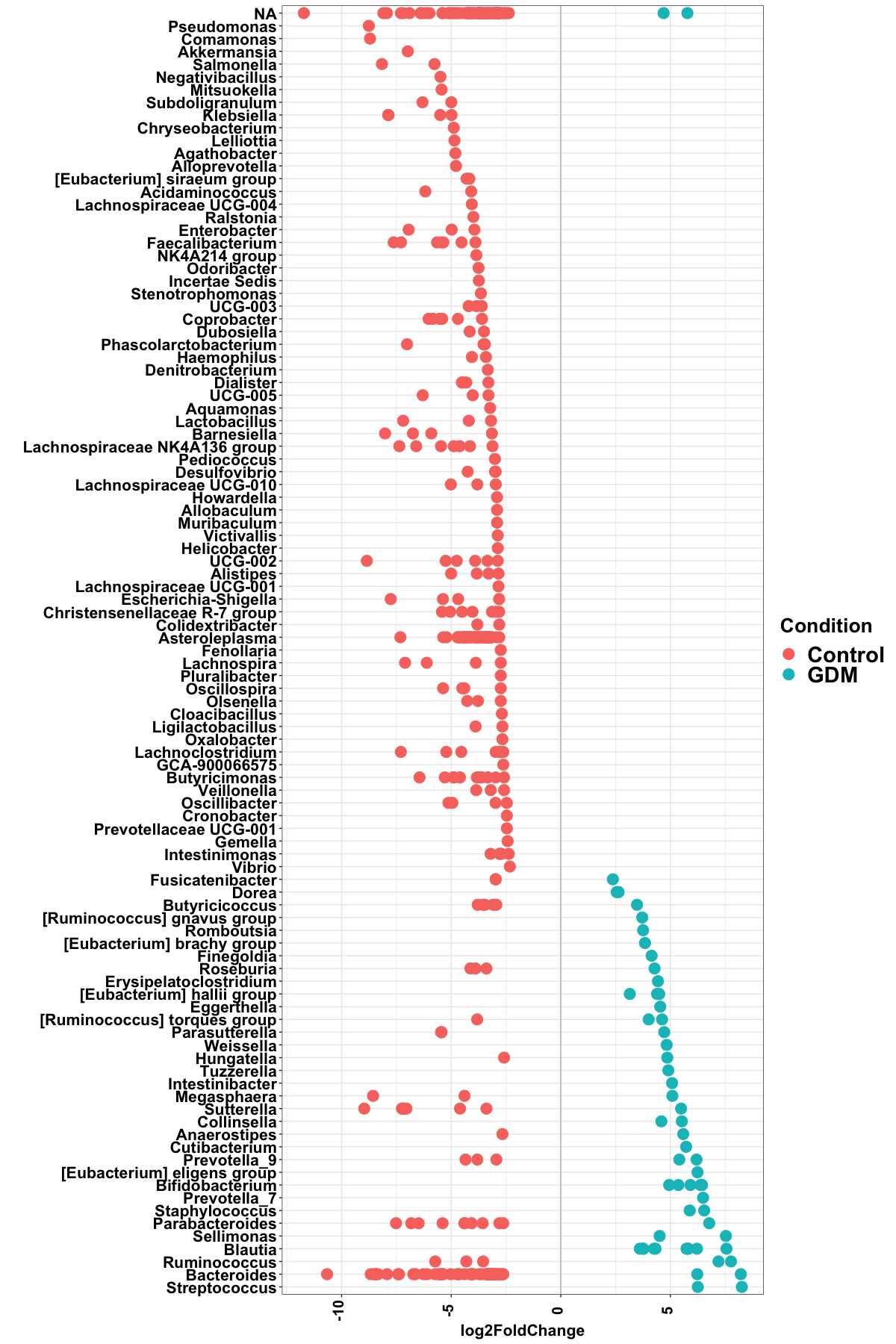

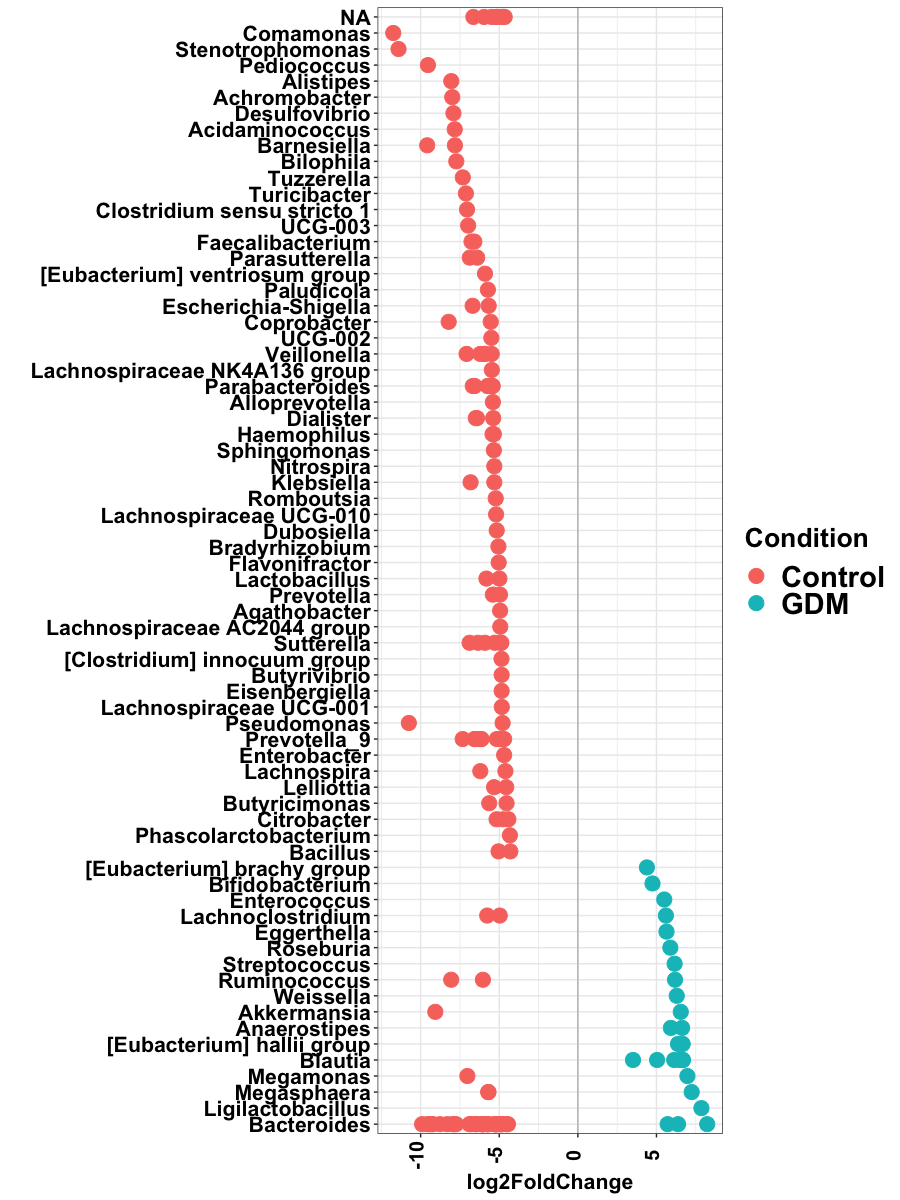

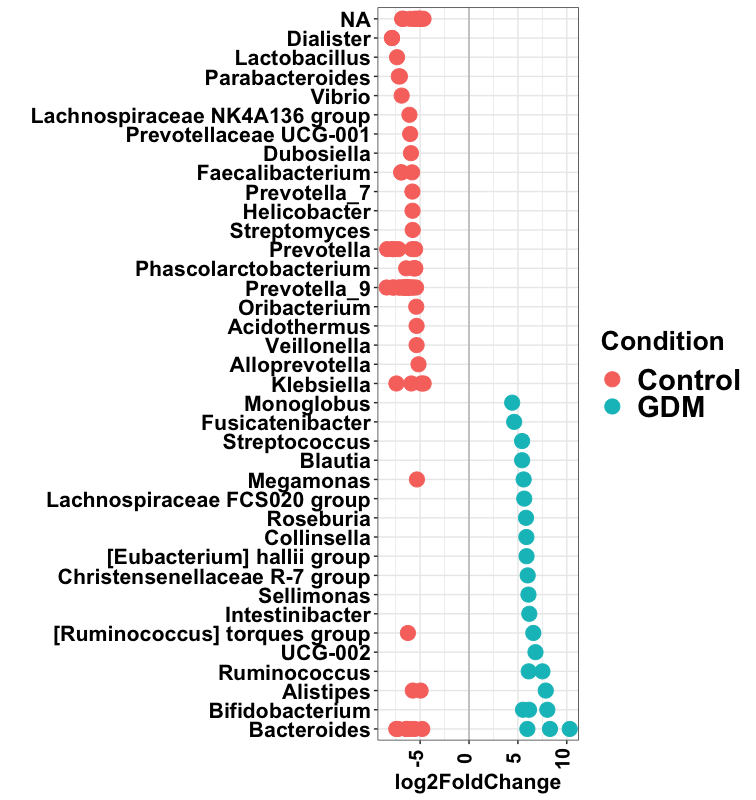


**Supplementary Fig. S2** Predictive metabolic functions derived from PICRUSt2 analysis of gut microbiome of GDM and control pregnant women. **(a)** PCA plot based on predictive metabolic functions; **(b)** Differentially abundant KEGG derived metabolic pathways.

**(a)**

**
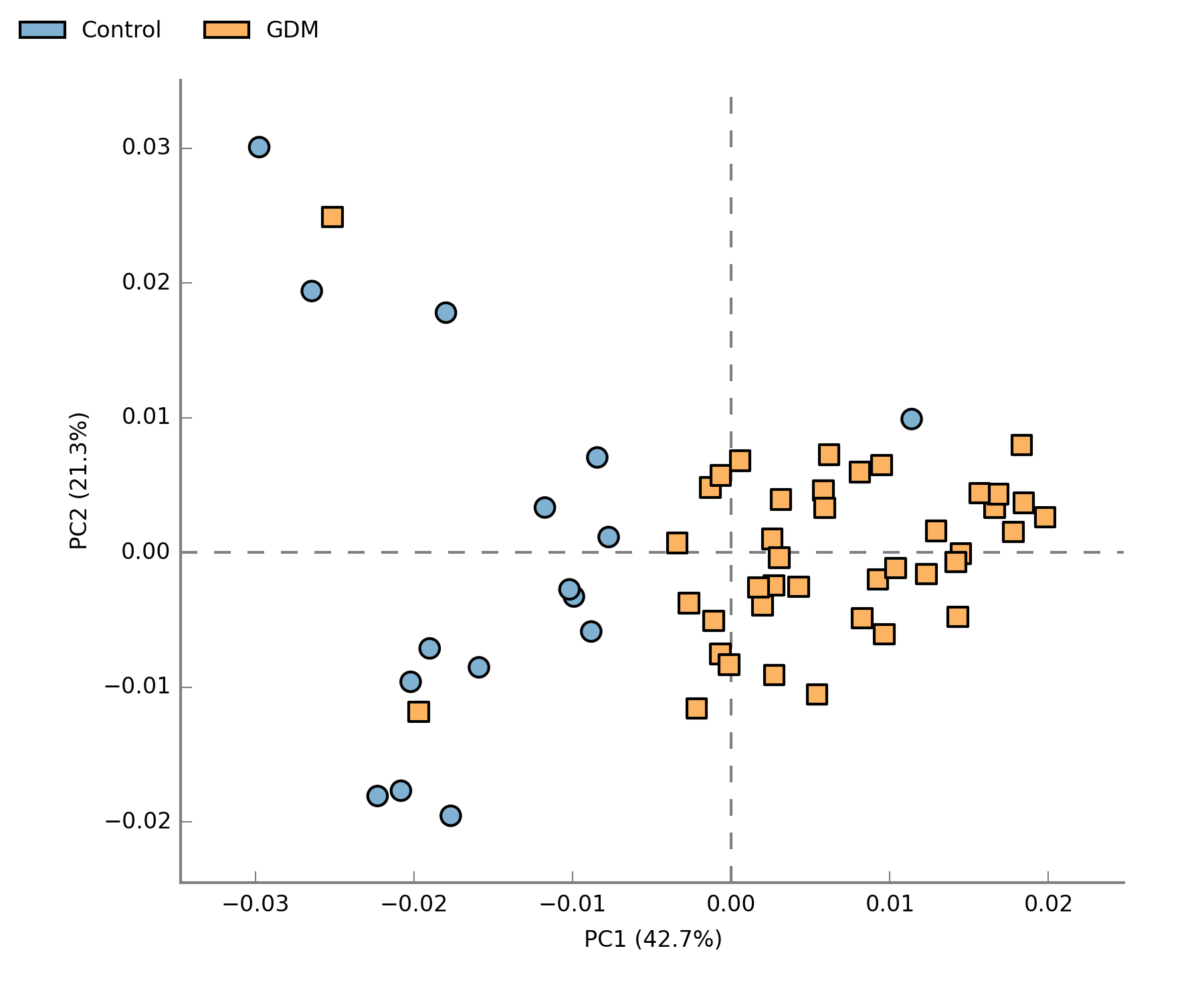
**

**
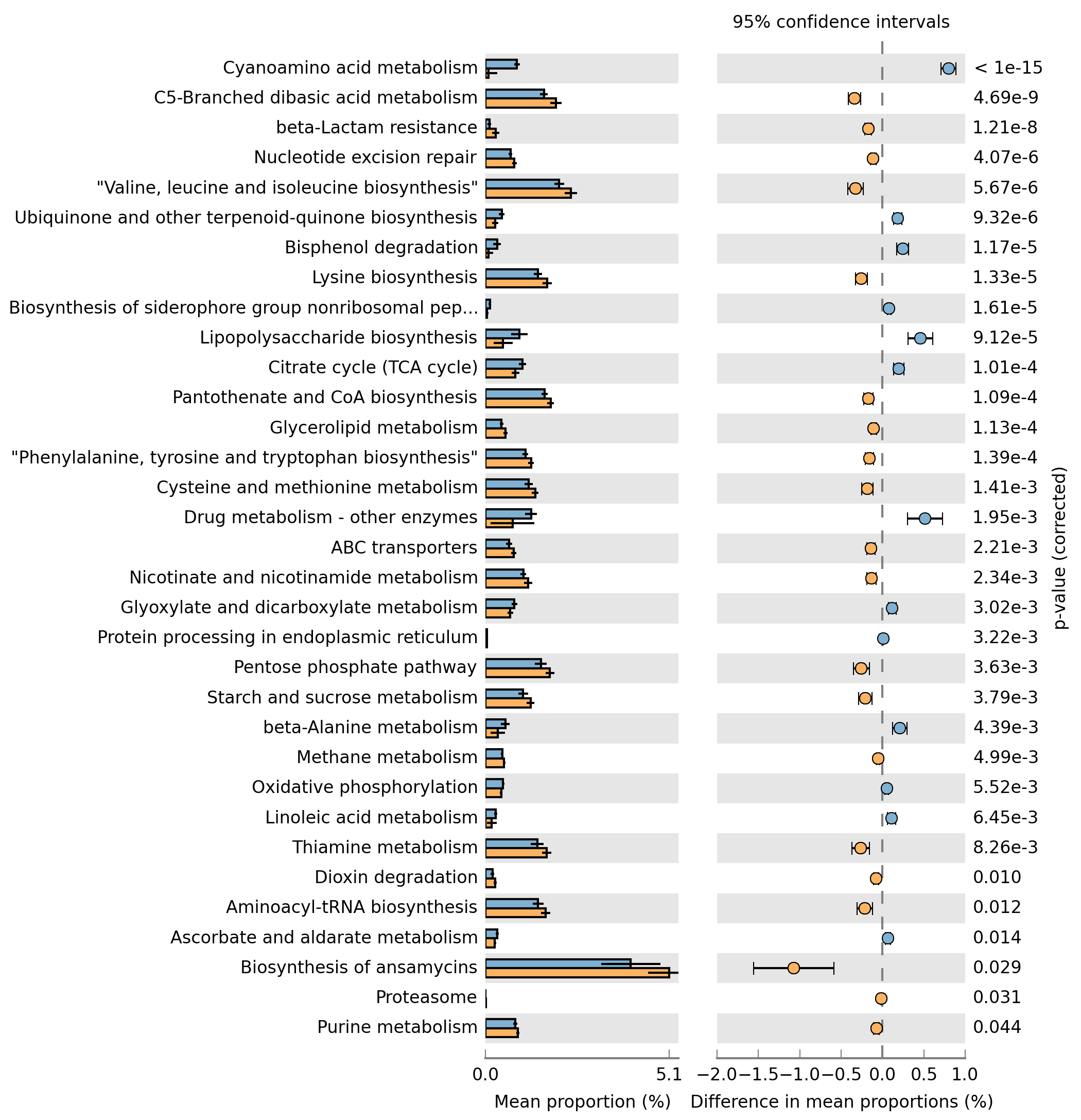
**

**(b)**

**
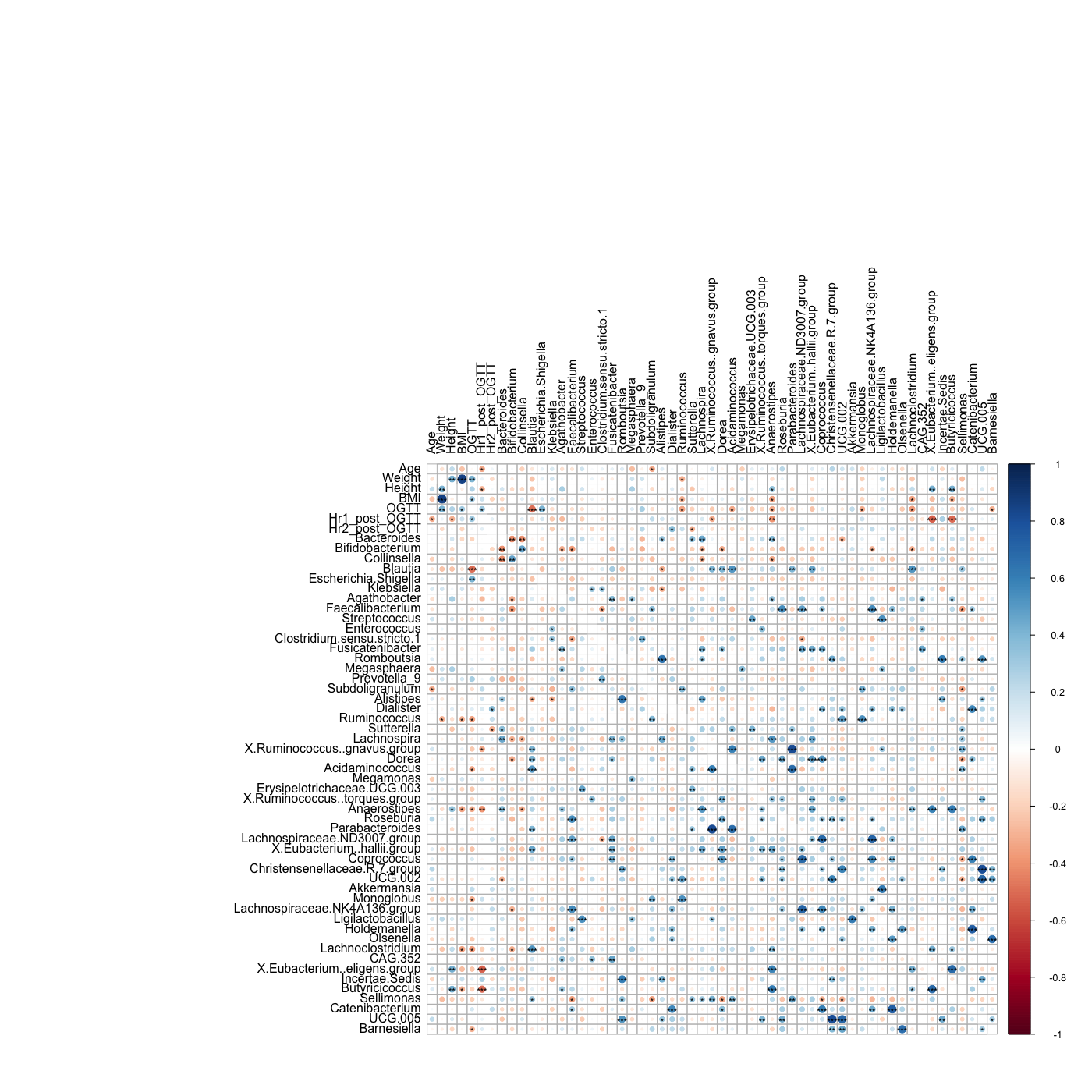
Supplementary Fig. S3** Heatmap based Spearman correlation association between clinical variables and bacterial genera at the time of diagnosis. *P<0.05; **P<0.01; ***P<0.001. Corrplot package v0.92 was used to generate this heatmap.
